# Structural Evolution of Bacterial Polyphosphate Degradation Enzyme for Phosphorus Cycling

**DOI:** 10.1002/advs.202309602

**Published:** 2024-04-29

**Authors:** Shang Dai, Binqiang Wang, Rui Ye, Dong Zhang, Zhenming Xie, Ning Yu, Chunhui Cai, Cheng Huang, Jie Zhao, Furong Zhang, Yuejin Hua, Ye Zhao, Ruhong Zhou, Bing Tian

**Affiliations:** ^1^ Institute of Biophysics College of Life Sciences Zhejiang University Hangzhou 310029 China; ^2^ Shanghai Institute for Advanced Study of Zhejiang University Shanghai 201203 China; ^3^ State Key Laboratory of Clean Energy Utilization Zhejiang University Hangzhou 310029 China; ^4^ Zhejiang Baima Lake Laboratory Co., Ltd Hangzhou 310029 China; ^5^ School of Physics Institute of Quantitative Biology Zhejiang University Hangzhou 310029 China; ^6^ Cancer Center Zhejiang University Hangzhou 310029 China

**Keywords:** ɑ‐linker, exopolyphosphatase, polyphosphate, structural evolution

## Abstract

Living organisms ranging from bacteria to animals have developed their own ways to accumulate and store phosphate during evolution, in particular as the polyphosphate (polyP) granules in bacteria. Degradation of polyP into phosphate is involved in phosphorus cycling, and exopolyphosphatase (PPX) is the key enzyme for polyP degradation in bacteria. Thus, understanding the structure basis of PPX is crucial to reveal the polyP degradation mechanism. Here, it is found that PPX structure varies in the length of ɑ‐helical interdomain linker (ɑ‐linker) across various bacteria, which is negatively correlated with their enzymatic activity and thermostability – those with shorter ɑ‐linkers demonstrate higher polyP degradation ability. Moreover, the artificial DrPPX mutants with shorter ɑ‐linker tend to have more compact pockets for polyP binding and stronger subunit interactions, as well as higher enzymatic efficiency (*k*_cat_/*K*_m_) than that of DrPPX wild type. In Deinococcus‐Thermus, the PPXs from thermophilic species possess a shorter ɑ‐linker and retain their catalytic ability at high temperatures (70 °C), which may facilitate the thermophilic species to utilize polyP in high‐temperature environments. These findings provide insights into the interdomain linker length‐dependent evolution of PPXs, which shed light on enzymatic adaption for phosphorus cycling during natural evolution and rational design of enzyme.

## Graphical abstract

This study demonstrates an interdomain linker‐based exopolyphosphatase (PPX) structural evolution in bacteria. The length of ɑ‐linker in PPX, which involves phosphate cycling, is varied among bacteria and has impacts on protein's conformation and quaternary structure, thus posing an impact on enzyme activity and thermostability. These results suggest a potential relationship between PPX structural evolution and bacterial environmental adaptability.

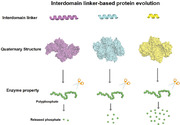

## 1 Introduction

Most of the living organisms ranging from prokaryote to eukaryote have evolved the physiological ability to accumulate and store phosphate (Pi) as the polyphosphate (polyP) granules in bacteria^[^ ^1^ ^]^ or the vacuoles in yeast and plants.^[^ ^2^, ^3^ ^]^ The polyP is a linear negatively‐charged inorganic polymer consisting of tens to hundreds of orthophosphate residues linked by high‐energy phosphoanhydride bonds, which could possibly be geographically produced by dehydration of phosphate rock at high temperatures and distributed mainly in volcanoes and deep‐oceanic steam vents.^[^ ^4^, ^5^ ^]^ As a ubiquitous molecule in living organisms, polyP might play an important role in the evolution of life as a plausible source of phosphorus and energy.^[^ ^5^, ^6^ ^]^ In bacteria, polyP is utilized in many cellular aspects including intracellular phosphate donor and phosphorylation agent.^[^ ^5^, ^6^ ^]^ PolyP is also involved in bacterial pathogenicity and persistence,^[^ ^7^, ^8^ ^]^ offering a strategy forantibiotic designing that targets the polyP metabolism pathway.^[^ ^9^ ^]^

In bacteria, polyP metabolism is controlled by polyP kinase (PPK) for polyP synthesis using ATP or GTP as the substrates, and exopolyphosphatase (PPX) for polyP catabolism.^[^ ^5^ ^]^The PPX hydrolyzes and releases the Pi progressively from the linear polyP chain. Thus, the degradation of polyP into Pi is primarily mediated by PPX,^[^ ^10^, ^11^ ^]^ which is important for Pi utilization and cycling in bacterial cells.^[^ ^12^, ^13^ ^]^ Bacterial PPXs share conserved catalytic domains 1 and 2 belonging to the catalytic N‐domain, and most members of the PPX family contain two additional domains 3 and 4 in the C‐terminal.^[^ ^14^ ^]^ From the few structurally characterized bacterial PPXs up to present including the PPX from *E. coli* (PDB: 1U6Z and 2FLO),^[^ ^14^, ^15^ ^]^ *Helicobacter pylori* (PDB: 6PC0)^[^ ^16^ ^]^ and *Aquifex aeolicus* (PDB: 2J4R) which contains only an N‐domain^[^ ^17^ ^]^, we observed a structural variety of the homologous PPXs. Understanding the structure basis of PPX is crucial to reveal polyP degradation mechanism. However, little is known about the molecular basis and structural evolution of PPX.

The structural variety of PPXs seems to imply a differential evolution path of PPX. In this study, we find that the length of the ɑ‐helical interdomain linker between the N‐domain and C‐domain in PPXs varies across bacterial species, which had a strong negative correlation with the enzymatic activity of PPX. Based on structure analysis of PPX and PPX‐polyP complexes, as well as molecular dynamics simulation and biochemical analysis, we elucidate the molecular mechanism underlying the interdomain linker length‐dependent activity of PPXs. Furthermore, we reveal the correlation of ɑ‐linker lengths with PPX activity and thermostability across species in the phylum Deinococcus‐Thermus, which illustrates the enzymatic adaption during natural evolution.

## 2 Result

### 2.1 The ɑ‐Helical Interdomain Linker Varies in Bacterial PPXs

**Figure 1:**
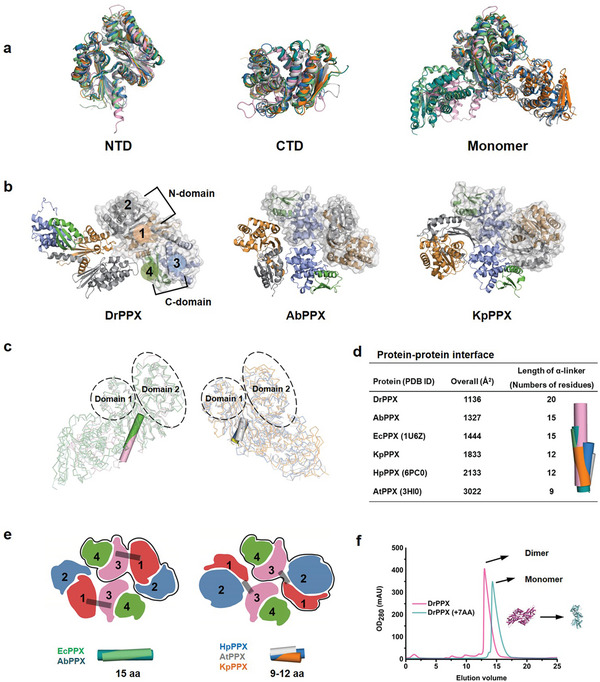
The length of the ɑ‐linker of PPX varies in bacteria. a) Superposition of N‐terminal domains (NTDs), C‐terminal domains (CTDs), and monomers of PPXs from different bacterial species. Pink: DrPPX from *Deinococcus radiodurans* (this study); Cyan: AbPPX from *Acinetobacter baumannii* (this study); yellow: KpPPX from *Klebsiella pneumoniae* (this study); green: EcPPX (PDB ID: 1U6Z) from *E. coli*; orange: HpPPX from *Helicobacter pylori* (PDB ID: 6PC0); gray: AtPPX from *Agrobacterium tumefaciens* (PDB ID: 3HI0). b) Crystal structures of PPX dimers from different species were determined in this study (the number indicates the respective domain). c) Ribbon representation of monomers of the aforementioned PPXs. The α‐linkers are highlighted as cylinders with different colors. d) Calculation of the dimeric interface between two monomers of PPXs using PDBePISA, with the values listed in the table. The α‐linkers are shown as cylinders with different colors. e) Diagram of the two types of PPX dimers as depicted by color blocks (the number in each block indicates the respective domain). The α‐linkers are shown as gray cylinders. f) Size exclusion chromatography of the wild‐type DrPPX and the DrPPX with extended α‐linker (+7AA).

Protein crystal structures of PPX homologs from different bacterial species, including DrPPX from *Deinoccocus radioduran*s, AbPPX from *Acinetobacter baumannii*, and KpPPX from *Klebsiella pneumoniae*, were determined and refined to resolutions of 1.8‐2.6 Å, respectively (crystallographic data together with the data collection and refinement statistics were summarized in **Table** **1**; Table S1, Supporting Information). While the KpPPX and AbPPX crystallized as a symmetric dimer, the DrPPX crystallographic structure exhibited an asymmetric homodimer (**Figure** **1**b). As shown in Figure 1a, the overall monomer structures of DrPPX, AbPPX, and KpPPX exhibited conserved domains at N‐ and C‐terminus and good superimposition with the known structures of EcPPX (PDB ID code 1U6Z) from *E. coli*, HpPPX (PDB ID code 6PC0) from *Helicobacter pylori* and AtPPX (PDB ID code 3HI0) from *Agrobacterium tumefaciens*. However, the PPX proteins adopt distinct dimer conformations with varying lengths of the α‐helical interdomain linker (designated ɑ‐linker) between the N‐ and C‐terminus. The AtPPX has a ɑ‐linker consisting of 9 amino acids (Gly298‐Ser306), the KpPPX and HpPPX have ɑ‐linkers of 12 amino acids (Ala296 – Gly307, Gly285‐Leu296, respectively), the EcPPX and AbPPX have ɑ‐linkers of 15 amino acids (Ala296 – Lys310), while the DrPPX has a ɑ‐linker of 20 amino acids (Ala300 – Ala319) (Figure 1c,d). The length variation of α‐linkers among the PPXs, which connected the N‐ and C‐terminal domains, suggested that the length of α‐linker may impact the relative orientations and interactions of the domains. The shorter α‐linker (AtPPX, KpPPX, and HpPPX) enabled the C‐terminal domain 3 to interact with domain 2 of the N‐terminal, while the longer α‐linkers (DrPPX, EcPPX, and AbPPX) facilitated the interaction of C‐terminal domain 3 with domain 1 and led to a relatively loose dimer formation (Figure 1c,e). Among the PPXs, the DrPPX which contains the longest α‐helical linker exhibited the smallest dimeric interface (1136 Å^2^) as calculated using the PDBePISA server, while the PPXs with shorter α‐linkers had a larger dimeric interface (Figure 1d). The result of regression analysis displayed a negative correlation between the dimer interface and α‐linker length (Figure S1, Supporting Information). Furthermore, we compared the superposition of the protein structures. It was observed that PPXs with similar lengths of α‐linkers presented similar types of dimers (Figure 1e; Figure S2, Supporting Information): the KpPPX, AtPPX, and HpPPX (9–12 AA in α‐linker) showed a compact dimer configuration, while the EcPPX and AbPPX (15 AA in α‐linker) displayed a relatively loose dimeric arrangement. Moreover, the mutant protein DrPPX (+7AA), containing an artificially extended α‐linker, could form a PPX structure with an extended α‐helix as predicted by Alphafold 2 (Figure S3a, Supporting Information). Using gel filtration chromatography, we observed a shift of the elution peak of DrPPX (+7AA) toward monomeric form compared with the DrPPX dimer (Figure 1f; Figure S3b, Supporting Information), indicating that the extended α‐linker could modulate the interactions between the domains, resulting in changes of quaternary structure. The results suggested that the length of α‐linker in PPXs varies among bacteria, and has an impact on interdomain actions and dimer configuration of PPX.

**Table 1:** Summary of PPX structures.

Structure name	protein	metal	ligand	PDB Code
DrPPX (WT)	DrPPX	Mg^2+^	SO_4_^2−^	8JGO
KpPPX	KpPPX	Mg^2+^, K^+^	‐	8JGW
AbPPX	AbPPX	‐	‐	8JGX
DrPPX (E114A)	DrPPX (E114A)	Mn^2+^	SO_4_^2−^	8JGT
DrPPX‐P2	DrPPX	Mn^2+^	PPi, PO_4_^3−^, SO_4_^2−^	8JGP
DrPPX‐P5	DrPPX	Mg^2+^	P5, SO_4_^2−^	8JGQ
DrPPX‐Pi	DrPPX	K^+^	PO_4_^3−^	8JGR
DrPPX‐NTD	DrPPX‐NTD	Na^+^	‐	8JGU

### 2.2 PolyP Hydrolysis Activity by PPX is Highly Correlated with the Length of ɑ‐linker

**Figure 2:**
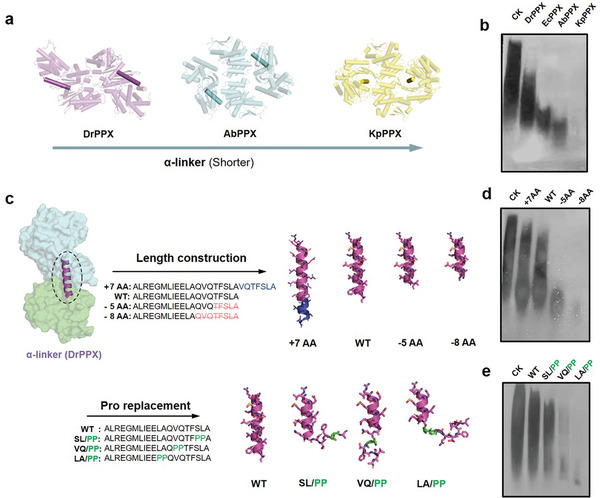
Negative correlation between the length of α‐linker and activity of PPXs. a) Structures of DrPPX, AbPPX, and KpPPX were shown with decreasing length of ɑ‐linker. The α‐linkers were highlighted. b) Urea‐PAGE analysis of polyP hydrolytic activity of the PPXs. CK, 100 µM polyP (P100) control in the absence of PPX; c) Diagram of length alteration of the ɑ‐linker in DrPPX. The structures of DrPPX variants were predicted using AlphaFold 2, and the ɑ‐linkers were extracted and displayed as cartoon cylindrical helices. The extended helix is shown in blue. The proline residue (Pro) replacement was depicted in green. d) Urea‐PAGE analysis of polyP hydrolytic activity of the DrPPX variants with adjusted α‐linker length (extension or truncation). WT, DrPPX; +7AA, DrPPX variant with an extension of α‐linker; −5AA and −8AA, DrPPX variants with shortened α‐linker; e) Urea‐PAGE analysis of polyP hydrolytic activity of DrPPXs with adjusted α‐linkers (proline replacement). For the Urea‐PAGE gel analysis of PPX activity, 20 µL reaction mixtures containing 100 µm polyP (P100), 2 mm MgCl_2_, and 2 µg PPX protein were used. The reaction was carried out at 37 °C for 15 min. Experiments b,d,e) were independently repeated three times with similar results, *n* = 3.

Next, we investigated whether the length of ɑ‐linker affects the enzymatic activity of PPXs. In terms of the polyP hydrolysis activity of the PPXs, the DrPPX with the longest ɑ‐linker exhibited the weakest activity, while the KpPPX with the shortest ɑ‐linker showed the strongest activity (**Figure** **2**a,b), demonstrating a strong negative correlation of ɑ‐linker length with PPX activity across the species.

To further investigate the relationship between ɑ‐linker length and enzyme activity, we constructed artificial variants of DrPPX with different ɑ‐linker lengths using two methods (Figure 2c). First, the C‐terminus of the ɑ‐linker was elongated or shortened. The results of polyP hydrolysis using Urea‐PAGE analysis demonstrated that the PPX activity increased with the shortening of ɑ‐linker but decreased with the extension of ɑ‐linker (Figure 2d). The DrPPX mutant (−8AA, deletion of QVQTFSLA in the ɑ‐linker) with a shorter ɑ‐linker showed a higher enzymatic efficiency (*k*_cat_/*K*_m_ = 48.38) than the DrPPX wild type (*k*_cat_/*K*_m_ = 2.28) (**Table** **2**). Second, we employed the proline substitution method to alter the length of the α‐linker. Proline (Pro) is commonly considered as a terminating amino acid in α‐helices because it introduces a kink to disrupt intrachain hydrogen bonds.^[^ ^18^ ^]^ We performed the amino acid substitutions with double proline at different positions within the α‐linker to create artificial variants of DrPPX with termination of the α‐linker: S317P/L318P (SL/PP), V313P/Q314P (VQ/PP), and L310P/A311P (LA/PP) (lower panel in Figure 2c). Structure prediction of these modified DrPPXs using Alphafold 2 showed that the length of ɑ‐linkers in the PPX variants was shorter compared to the wild‐type DrPPX (Figure. 2c). The polyP hydrolysis assay demonstrated a substantial increase of the PPX activity by interrupting the ɑ‐linker at a position close to the N‐terminus. Among the variants, the DrPPX (LA/PP) with the shortest ɑ‐linker exhibited the strongest activity (Figure 2e) with an increased *k_cat_/K_m_* (Table 2). These results confirmed the negative correlation between the polyP hydrolysis activity and the length of the α‐linker in PPX.

**Table 2:** Biochemical characterization of DrPPX wild‐type and variants.

Protein [variant]	*k*_cat_ [s^−1^]^a)^	*K*_m_ [µm]^a)^	*R* ^2^	*k*_cat_/*K*_m_ [µM s^−1^]
DrPPX(WT)	37.95 ± 3.05	16.68 ± 5.84	0.94	2.28
N11A	15.61 ± 1.3	20.98 ± 6.51	0.93	0.74
S12A	13.37 ± 1.6	74.86 ± 23.75	0.94	0.18
H14A	16.04 ± 1.13	44.22 ± 9.75	0.97	0.36
K35A	18.20 ± 2.8	64.99 ± 22.28	0.93	0.28
S141A	24.58 ± 1.42	25.58 ± 5.15	0.97	0.96
R271A	9.95 ± 1.93	79.44 ± 39.63	0.86	0.13
D7A	1.75 ± 0.27	35.10 ± 17.40	0.83	0.05
E114A	2.11 ± 0.47	46.17 ± 22.86	0.81	0.05
D136A	4.53 ± 0.55	44.36 ± 13.99	0.93	0.1
E143A	3.64 ± 1.03	84.23 ± 41.04	0.85	0.04
NTD	17.08 ± 2.05	109.1 ± 26.03	0.97	0.16
+7AA	16.36 ± 1.03	42.04 ± 7.77	0.98	0.39
−5AA	146 ± 13.1	12.4 ± 3.4	0.90	11.77
−8AA	277.7 ± 12.7	5.74 ± 1.28	0.95	48.38
SL/PP	49.8 ± 4.05	14.89 ± 4.27	0.92	3.34
VQ/PP	123.3 ± 8.0	11.86 ± 3.01	0.94	10.40
LA/PP	196.7 ± 8.1	4.96 ± 1.05	0.96	39.66

^a)^ data are presented as mean ± SD, *n* = 3

### 2.3 Molecular Interaction of PPX with PolyP

**Figure 3:**
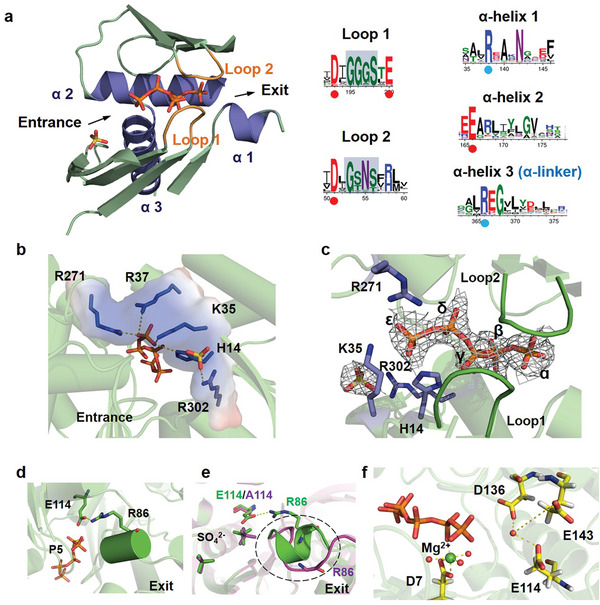
Molecular interaction between PPX and polyP (P5). a) The Catalytic core region of DrPPX with polyP (P5) (left panel) and the highly conserved amino acids in the catalytic core region of PPXs among various species (124 species) were marked by dots (right panel). PolyP entrance and exit were indicated by arrows. b) PolyP entrance and binding sites in DrPPX. Positively charged residues were shown as blue sticks. c) DrPPX–polyP (P5) interaction. The binding loops (Loop1 and Loop2) were highlighted with green color, positively charged residues are shown as blue sticks, and P5 was represented by brown sticks. The electron density of P5 was depicted in gray, with the refined 2Fo‐Fc map contoured at 1σ. d) The interaction between E114 and R86 in DrPPX‐P5 complex. The ɑ‐helix 1 was highlighted as a cylinder. e) Comparison of conformations between DrPPX and DrPPX (E114A). The ɑ‐helix 1 transformed into a loop when arginine (E114) was mutated to alanine (A114). The loop was highlighted in magentas. f) Conserved acidic amino acids interacting with Mg^2+^ and water molecules in DrPPX. The Mg^2+^ ion was located between two loops, as determined by molecular docking and molecular dynamics simulation. Three water molecules (red spheres), the oxygen atoms of αP and βP, and D7(Asp7) formed a coordinate bond with Mg^2+^. E114, D136, and E143 interacted with a water molecule.

To better understand the underlying molecular mechanism of ɑ‐linker length‐dependent PPX activity, we further investigated the structural basis of polyP cleavage by PPX. The exopolyphosphatase belongs to the ASKHA (Acetate and Sugar Kinases, Hsp70, Actin) superfamily protein, which utilizes a common fold for binding and catalyzing hydrolysis of ATP phosphoanhydride.^[^ ^14^ ^]^ The PPX's NTD structure with catalytic core is highly conserved (Figure 1a) and features a mixed five‐stranded β‐sheet and lateral helical segments (**Figure** **3**a). The catalytic core of DrPPX with P5 (polyP, *n* = 5) is shown in Figure 3a. Sequence alignment of PPXs from 124 species encompassing most of the phyla of bacteria revealed high conservation of residues in two loops (Loop 1 and Loop 2) and three α‐helices (ɑ‐helix 1, 2, and 3) of the PPX catalytic center (Figure 3a). Notably, Loop 1 (β1 loop) contains a conserved aspartate (D136 in DrPPX) and glutamate (E143 in DrPPX), while Loop 2 (β2 loop) has a conserved aspartate (D7 in DrPPX). The ɑ‐helix 1 and ɑ‐helix 2 contain highly conserved arginine and glutamate (R86 and E114 in DrPPX), respectively, while the ɑ‐helix 3 (the ɑ‐linker) has a conserved motif REG (Figure 3a).

In the DrPPX apo crystals grown in the presence of ammonia sulfate (Table 1), sulfate ions were observed in the cleft of the N‐terminal domain of the apo structure of DrPPX (Figure S4, Supporting Information). For the crystal of DrPPX grown in the presence of phosphate solutions (NaH_2_PO_4_ and K_2_HPO_4_), four phosphate ions were located in the polyP‐binding area (Figure S4, Supporting Information). To investigate the catalytic mechanism of PPX, we incubated DrPPX with polyP (P20, *n* = 20) to obtain DrPPX‐polyP complex structure. Along with the observed single phosphates from polyP degradation, a well‐defined electron density of a P5 product (polyP, *n* = 5) was observed in the cleft of the N‐terminal domain of the DrPPX‐polyP complex in the presence of 2 mm Mg^2+^, whereas a further hydrolytic product pyrophosphate (PPi) was observed in the presence of 8 mm Mn^2+^ (Figure S4, Supporting Information). In the DrPPX‐P5 complex structure, the polyP was tightly bound in the inner cavity of PPX (Figure 3b,c), interacting with four positively charged residues (H14, K35, R37, and R271). Additionally, R302 was bound to a sulfate near the entrance of polyP. Except for R37, these residues were highly conserved within PPXs (Figure S5, Supporting Information), thus they might play an important role in attracting polyP into the catalytic center. Alanine (A) substitutions of these residues diminished the enzymatic activity of DrPPX, as indicated by decreased *k_cat_* (Table 2). In the DrPPX‐P5 complex, product P5 was bound between Loop 1 and Loop 2 (Figure 3c), probably through hydrogen bonds with hydroxyl groups from conserved residues in the loops (Figure 3a). The ɑ‐phosphate of P5 was coordinated by T10, G140, and S141 from DrPPX, and the β‐phosphate interacted with residue T82; while the γ‐phosphate was bound to the backbone of residues T10, N11, and G139. The δ‐ and ε‐phosphates interacted with Loop1 (N11, S12, G139) and the positively charged residues (R271 and H14) from DrPPX. Alanine substitutions of these residues (T10, N11, S12, and S141) diminished the enzyme activity of DrPPX at various levels (Table 2), which was consistence with structural analysis. On the other side of the polyP binding tunnel, E114 interacted with both P5 and a conserved positively charged residue R86 which was located at ɑ‐helix 1 (ɑ1) (Figure 3d). We also determined the crystal structure of the E114A mutant of DrPPX. In the structure of the E114A mutant, the ɑ‐helix 1 adopted a shifted loop conformation (Figure 3e), and the E114A mutant lost most of its enzymatic activity (Table 2), suggesting that the interaction between E114 and R86 might be crucial for the catalytic function of DrPPX.

In a previously solved structure of the HpPPX‐Pi‐Mg^2+^ complex, the interaction of Asp9 residue (corresponding to D7 in DrPPX) with Mg^2+^ was necessary for PPX activity.^[^ ^16^ ^]^ Moreover, the catalytic region of the ATPase domain of Hsp70 which shares high similarity with that of PPX needs a magnesium ion to interact with the β‐phosphate of ATP.^[^ ^19^ ^]^ However, magnesium ion was not observed in the active site of the DrPPX crystal structure obtained in this study. To explore the potential binding of a magnesium ion in the polyP‐binding pocket, we performed docking and molecular dynamics simulation analysis as detailed in Supporting Information Text. The missing regions (residue 384–401) of the DrPPX crystal structure were modeled using Alphafold 2 (Figure S6, Supporting Information). Molecular docking was performed based on the positioning of Mg^2+^ in HpPPX and Hsp70. The results of molecular docking and molecular dynamics simulation revealed that Mg^2+^ was stabilized by interactions with βP of polyP, the Asp (D7) from PPX, and three surrounding water molecules (Figure 3f; Figures S7 and S8, Supporting Information), which was similar to the Mg^2+^‐ATP binding mode (βP‐Mg^2+^) in Hsp70.^[^ ^19^ ^]^ Mutation of D7 into Ala resulted in a substantial decrease in enzymatic activity (Table 2). Therefore, the presence of Mg^2+^ ion in proximity to the βP of polyP (Figure S7b, Supporting Information) was likely a more stable binding site. Moreover, crystal structures of PPXs exhibited that three conserved residues (E114, D136, and E143 in DrPPX) interacted with a water molecule (Figure S9, Supporting Information), which is consistent with the molecular dynamics simulation results (Figure 3f). Mutations of these amino acids into Ala also lead to a decrease in enzymatic activity (Table 2). These results elucidated the molecular interaction of PPX with polyP, which is helpful in understanding the mechanism of PPX activity.

### 2.4 Molecular Mechanism of ɑ‐Linker Length‐Dependent PPX Activity

**Figure 4:**
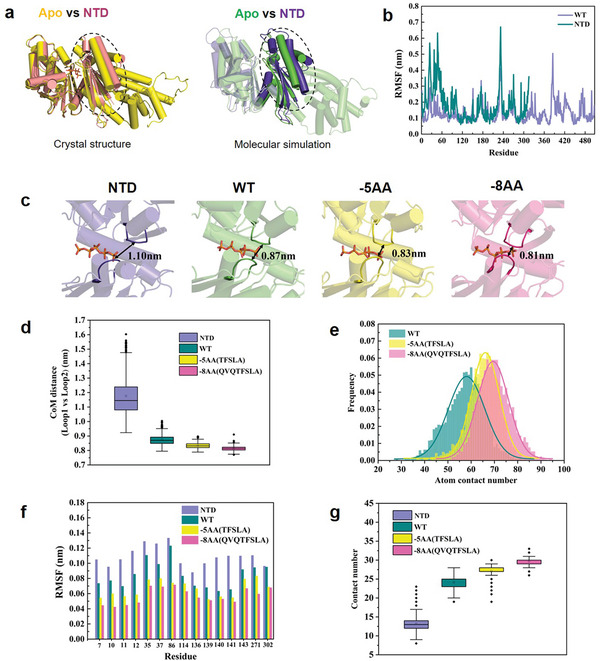
Molecular mechanism of ɑ‐linker length‐dependent PPX activity. a) Structural comparison of apo DrPPX and NTD of DrPPX based on crystal structures and molecular simulation, respectively. b) Root mean square fluctuation (RMSF) of apo‐DrPPX and NTD of DrPPX determined by molecular simulation. c) PolyP binding pocket of DrPPX and its variants. WT, DrPPX wild type; NTD, C‐terminal truncation mutant of DrPPX; −5AA and −8AA, DrPPX variants with shortened α‐linker. The loops (Loop 1 and Loop 2) are highlighted. d) The center of mass (CoM) distance between Loop 1 and Loop 2 in the polyP binding pocket of NTD, WT, −5AA, and −8AA. Data were obtained from the last 100 ns of three independent trajectories. e) Frequency distribution of the atom contact number between the C‐terminus and N‐terminus of WT, −5AA and −8AA. Data were obtained from the last 100 ns of three independent trajectories. f) RMSFs of key amino acids in the polyP binding pockets of NTD, WT, −5AA, and −8AA. g) The atom contact number of polyP with Loop 1 and Loop 2 in the polyP binding pockets of NTD, WT, −5AA, and −8AA. Data were obtained from the last 100 ns of three independent trajectories.

Based on the interaction analysis of PPX and polyP, we next investigated the molecular mechanism of the ɑ‐linker length‐dependent activity of PPXs. First, we investigated the interaction of C‐terminal and N‐terminal in PPXs. The C‐terminal domains of many proteins have been found to contribute to protein function or enzyme activity.^[^ ^20^, ^21^, ^22^ ^]^ The C‐terminal domains of EcPPX have been shown to impact its polyP hydrolytic activity.^[^ ^14^ ^]^ We determined the crystal structure of the N‐terminal domain of DrPPX (Table 1). Superposition of apo‐ and NTD structures of DrPPX revealed that truncation of the C‐terminal led to an enlargement of the cleft between domain 1 and domain 2 of the N‐terminal (**Figure** **4**a). This suggested that the C‐terminal probably exerted a force on the N‐terminal active regions. Next, molecular dynamics simulation was used to further investigate the impact of the C‐terminal domain on the overall protein structure. The result indicated that C‐terminal domains might “pushed” domain 1 toward domain 2 (Figure 4a), which was consistent with the comparison of apo DrPPX and NTD crystal structures. The root‐mean‐square fluctuation (RMSF) values of residues in domain 1 (1–120 AA) of the NTD were higher than those of apo DrPPX (Figure 4b), indicating that residues in domain 1 of the NTD tend to be more unstable, which may affect polyP binding and catalysis. Moreover, the NTD of DrPPX exhibited a substantially decreased enzymatic activity compared to that of full‐length DrPPX (Table 2).

Using the wild type (WT) of DrPPX as a template, we simulated DrPPX mutants with different α‐linker lengths, referred to as −5AA, and −8AA, respectively (Figure S10, Supporting Information). Figure S11 (Supporting Information) showed the final stable protein structures of −5AA and −8AA. To obtain the simulated structures, distance restraints were imposed on the CTD and NTD of DrPPX, and a microsecond molecular dynamics simulation was conducted to allow them to move to a reasonable position (Figure S12a, Supporting Information). Subsequently, the distance restraints of the CTD and NTD were removed, and another microsecond molecular dynamics simulation was performed until the root‐mean‐square deviation (RMSD) reached stability (Figure S12b, Supporting Information). Figure 4c illustrates the simulation details of polyP binding pockets of NTD, WT, −5AA, and −8AA. First, we calculated the center of mass (CoM) distance between Loop 1 and Loop 2 in the polyP‐binding pocket of NTD, WT, −5AA and −8AA. Compared with the NTD, the CoM distance between the loops in the binding pockets of WT, −5AA, and −8AA gradually decreased (Figure 4c,d). The two loops approached closer in −8AA as compared with that of WT. Thus, the −8AA exhibited a tighter binding pocket compared with that of WT (Figure S13, Supporting Information). Second, we calculated the distributions of atom contact numbers between the NTD and CTD of WT, −5AA, and −8AA on the lasting 100 ns trajectories, respectively (Figure 4e). Here, any heavy atoms of the NTD and the CTD were considered to be in contact if the distance between them was less than 5 Å, and the number of heavy atoms of CTD that were in contact was counted. It was shown that the atom contact number between NTD and CTD increased with the decrease of α‐linker length, suggesting that the contact between NTD and CTD became closer with the shortening of the α‐linker. To assess the binding stability of polyP, we also calculated the RMSFs of key residues (mentioned in Figure 3) in the pocket. Figure 4f shows that the RMSFs of the key residues decreased in the order of −8AA < −5AA < WT < NTD, indicating the increase of polyP binding stability of PPX with shortened α‐linker, which may be more favorable for the catalytic hydrolysis of polyP. Furthermore, we analyzed the atom contact number of polyP with the two loops (Figure 4g). Here, polyP and any heavy atoms of Loop1 and Loop2 were considered to be in contact if the distance between them was less than 5 Å, and the number of heavy atoms of the loops that were in contact was counted. It can be seen that the atom contact number increased in the order of −8AA > −5AA > WT > NTD, indicating that the contact between polyP and the two loops gradually increased (Figure 4g), which was consistent with the results of Figure 4c,d. Thus, the shorter α‐linker could cause a closer interaction between the C‐terminus and N‐terminus, leading to a stronger interaction and binding of polyP with the active amino acids of the PPX. These results confirmed that the α‐linker in PPX plays a crucial role in enzyme activity and might be related to the evolution of PPXs.

### 2.5 The α‐Linker Dependent PPX Evolution in Deinococcus‐Thermus Phylum

**Figure 5:**
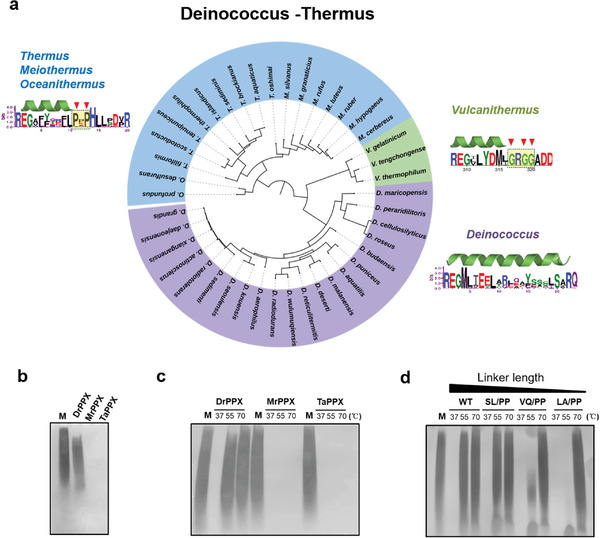
The α‐Linker length‐dependent activity and thermostability of PPX homologs in the Deinococcus‐Thermus. a) Phylogeny of the bacterial PPXs in Deinococcus‐Thermus (42 species). Phylogenetic analysis was performed using MEGA. The unrooted phylogenetic tree was visualized using ITOL. Taxonomy was annotated accordingly. The structures of PPXs were predicted using the AlphaFold 2, and the α‐linker sequences were extracted and shown in the cartoon. The sequences containing the α‐linkers, which were started by the conserved REG motif, were analyzed using WebLogo. Conserved amino acids that potentially interrupt the α‐linker were indicated by red triangles. b) Urea‐PAGE analysis of PPX activities. Reactions were performed for 15 min at 37 °C. M: polyP (P100); DrPPX: PPX from *Deinococcus radiodurans*; MrPPX: PPX from *Meiothermus ruber*; TaPPX: PPX from *Thermus aquaticus*. c) Urea‐PAGE analysis of PPX activities at different temperatures. Reactions were performed for 30 min at 37, 55, or 70 °C. d) Urea‐PAGE analysis of activities of PPX with modified α‐linker by proline substitutions at different temperatures. M: polyP (P100); WT: DrPPX; SL/PP: DrPPX (SL/PP); VQ/PP: DrPPX (VQ/PP); LA/PP: DrPPX (LA/PP). Reactions were performed for 30 min at 37, 55, or 70 °C. Experiments b–d) were independently repeated three times with similar results, *n* = 3.

Bacteria from the phylum Deinococcus–Thermus are known for their tolerance to various stresses including radiation, oxidation, desiccation, and high temperature. The phylum comprises two distinct groups of organisms and is classified into two orders: *Deinococcales* and *Thermales*. The order *Thermales* encompasses five genera (*Thermus*, *Meiothermus*, *Marinithermus*, *Oceanithermus*, and *Vulcanithermus*), while the order *Deinococcales* consists of three genera (*Deinococcus*, *Deinobacterium*, and *Truepera*). Thermus species are either thermophilic or hyperthermophilic, while most Deinococcus species are nonthermophilic but exhibit resistance to extreme DNA‐damaging stress including ionizing radiation and desiccation.^[^ ^23^, ^24^ ^]^ PPX homologs are widely distributed across the Deinococcus–Thermus species with distinct environmental adaptions. We performed a phylogenetic analysis of the PPX/GppA family homologs from 42 species of the Deinococcus‐Thermus phylum using the MEGA program (**Figure** **5**a). Conservative analysis of the ɑ‐linker revealed a highly conserved motif “REG” at the beginning of the ɑ‐linker N‐terminal (Figure 5a). In the *Deinococcus* bacteria, the predicted ɑ‐linker length was averaged in 20 amino acids. However, the ɑ‐linker of PPXs in the genera of *Thermus*, *Meiothermus*, and *Oceanithermus* was blocked by a conserved “Pro‐X‐Pro” motif at the 11th amino acid position from “REG.” On the other hand, the ɑ‐linker of PPXs in the genera of *Vulcanithermus* was blocked by a glycine‐rich motif “GRGG,” which tends to form loops (Figure 5a). The results indicated that the thermophilic species from the order *Thermales* possess PPXs with short ɑ‐linkers of ≈10 AA‐length, while the species from *Deinococcus* have PPXs with long ɑ‐linkers of ≈20 AA‐length (Figure 5a; Figure S14, Supporting Information).

To further verify the ɑ‐linker length‐dependent PPX activity, we expressed and purified the PPX homologs from *Meiothermus ruber* (MrPPX) and *Thermus aquaticus* (TaPPX) for enzyme activity analysis. The results showed that the MrPPX and TaPPX exhibited much higher polyP hydrolysis activity compared with the DrPPX (Figure 5b). Considering that the thermophilic bacteria within *Thermales* usually inhabit high‐temperature environments, and possess PPX homologs with short ɑ‐linkers (Figure 5a), we hypothesized that the ɑ‐linker length may have an effect on the thermostability of PPX. The MrPPX and TaPPX retained their catalytic ability at high temperatures (70 °C) to completely hydrolyze polyP, indicating their thermostability and efficient utilization of polyP; in contrast, the activity of DrPPX from the non‐thermophilic *D. radiodurans* was almost diminished at 55 and 70 °C (Figure 5c). Furthermore, the DrPPX mutants (VQ/PP and LA/PP) with shortened ɑ‐linkers still exhibited partial polyP hydrolysis activity at 55 °C (Figure 5d) compared with the wild‐type DrPPX, suggesting that shortening of the ɑ‐linker increased the stability of PPX at high temperatures. We measured the *T*_m_ value of PPXs using the Differential Scanning Calorimetry. The *T*_m_ value of MrPPX, TaPPX, and DrPPX (LA/PP) was higher than that of DrPPX (Figure S15, Supporting Information), confirming that the PPXs with short ɑ‐linkers have relatively high thermostability. Taken together, these findings suggested that the interdomain ɑ‐linker plays an important role in the enzymic function and temperature adaptability of PPXs, which might enable the maintenance of intracellular Pi homeostasis for bacteria to adapt to distinct environments.

## 3 Discussion

By analyzing the crystal structures of PPXs from different bacterial species, we discovered that the α‐linkers vary in length among the PPXs. The PPXs with distinct α‐linker lengths exhibited different dimer configurations, and the compactness of the dimer interfaces was negatively correlated with the linker length (Figure 1; Figure S1, Supporting Information). Previous studies have shown that an interdomain linker played an important role in the self‐oligomerization of the adeno‐associated virus Rep protein (AAV‐2 Rep68).^[^ ^25^ ^]^ Similarly, mutations or extensions in the interdomain linker of antibody light chain variants can regulate domain orientation.^[^ ^26^ ^]^ Interdomain linkers can also regulate the autophosphorylation of histidine kinase.^[^ ^27^ ^]^ The length of the “rigid bridge”‐like α‐linker that connects the domains in PPXs varies across bacterial species, suggesting the correlation between the α‐linker and enzymic evolution.

Structural change usually has impacts on the function of enzymes, even when the changes occur in regions distant from the catalytic active center.^[^ ^28^, ^29^, ^30^ ^]^ Among the PPXs from different bacterial species, those with shorter α‐linkers exhibit stronger enzyme activities (Figure 2b). This negative correlation was also identified using artificially modified PPXs with extended or shortened α‐linkers: the shorter the linker, the stronger the activity of PPX. Our results revealed that polyP interacted with two conserved loops (Loop 1 and Loop 2) and positively charged residues in the polyP binding pocket. Generally, substrate binding capacity has effects on enzyme catalysis.^[^ ^31^, ^32^ ^]^ Moreover, the crystal structures of the DrPPX complex with the pyrophosphate and P5 products provided insights into the hydrolysis process of polyP by PPX. The positively charged surface near the entrance hole (Figure S5, Supporting Information) might exert a Coulombic force on the negatively charged polyP. These indicated that polyP might be initially attracted by the positively charged entrance, then translocated into the cleft formed by two loops, followed by subsequent hydrolysis.

Our results of molecular simulation demonstrated that shortening of the α‐linker could increase the interactions between the C‐terminus and N‐terminus, thereby reducing the center of mass distance between the two loops involved in polyP binding and increasing the atom contact number between polyP and the binding pocket. These findings explain the molecular mechanism behind the interdomain linker length‐dependent PPX activity. The findings opened an avenue for structure‐based designing and directed evolution of PPX using rational design methods, which are effective approaches to improving catalytic efficiencies of both natural and artificial enzymes.^[^ ^28^ ^]^ Previous studies on the structural and conformational variants of proteins during natural evolution provide valuable insights for enzyme rational designing and understanding enzyme evolution.^[^ ^29^, ^30^ ^]^

We verified the relationship of ɑ‐linker length with the activity of PPXs in the extremophilic phylum Deinococcus‐Thermus. Following the evolution from their common ancestor, the *Thermus* and *Deinococcus* lineages have taken divergent paths toward their distinct lifestyles. The thermophilic bacteria from *Thermus*, *Oceanithermus*, and *Vulcanithermus* tended to have PPXs with shorter ɑ‐linkers, higher polyP hydrolysis activity, and thermostability, in contrast with those of the nonthermophilic bacteria from *Deinococcus*. The formation of a short ɑ‐linker secondary structure might be an evolutionary strategy for thermophilic bacteria to make a compact protein to overcome protein denaturation caused by high temperatures. The thermophilic bacteria from *Thermus*, *Oceanithermus*, and *Vulcaniibacterium* living in high‐temperature environments, e.g., hyperthermal vents and volcanoes, which were potentially rich in polyP,^[^ ^4^, ^5^ ^]^ may have evolved PPXs with short ɑ‐linkers to efficiently utilize polyP as a phosphorus source. It was reported that extracellular polyP could enter cells in the form of nanoparticles with metal ions.^[^ ^33^ ^]^ For the nonthermophilic bacteria from *Deinococcus* with PPXs containing relatively longer ɑ‐linkers, these bacteria might not require PPXs with high activity since they usually originate from environments with extreme radiation or desiccation (desert)^[^ ^34^ ^]^ where probably have not a large amount of polyP. On the other hand, *Deinococcus* bacteria could achieve intracellular polyP homeostasis and response to oxidative stress through expression regulation of the PPX.^[^ ^35^ ^]^ These suggested a differential evolution of PPXs, which support different bacterial species to adapt to distinct environments.

During bacterial evolution, a natural mutation of amino acid might alter the ɑ‐linker length and enzyme activity of PPX, which is involved in the regulation of phosphorus cycling. Interaction of microorganisms with abiotic or biotic factors could alter microbial enzymic activity as well as phosphorus bioavailability.^[^ ^36^ ^]^ The natural evolution of PPX might be the outcome of interactions of bacteria with biotic or abiotic stresses. Moreover, the ɑ‐linker in PPXs might be a site of regulation in vivo. Amino acids in the ɑ‐linker might be modified or changed by a potential enzyme or gene‐editing system and result in the alternation of the α‐linker length. Thus, the evolution of PPX with altered α‐linker length through yet‐to‐be‐determined mechanisms in bacteria could achieve polyP metabolism regulation, which is important in many cellular aspects. PolyP is involved in bacterial pathogenicity through interfering with the innate host defense to infection.^[^ ^37^ ^]^ PPXs with different α‐linker lengths and activities in pathogenic bacteria might also play important roles in bacterial adaptation to the host immune system and the subsequent pathogenicity by controlling intracellular polyP level. Thus, it deserves further study on the evolution and regulation mechanism of PPX with varied α‐linker lengths in connection with multiple cellular aspects including pathogenicity deserves further study. Also, the evolutionary relationship between the ɑ‐linker and PPX structure should be further explored in the entire bacterial kingdom.

In sum, the present study provides findings on an interdomain linker length‐dependent structural evolution of PPXs underlying phosphorus metabolism and cycle in bacteria. PolyP serves as an evolutionarily ancient source of phosphorus for living organisms. Our results will contribute to understanding the polyP metabolism in bacteria, and shed new light on the enzymatic adaption for phosphorus cycling during natural evolution.

## 4 Experimental Section

### Cloning, Expression, and Protein Purification

Gene encoding PPX homologs was amplified from the genome of *D. radiodurans* R1, *E*. *coli* K12, *A*. *baumannii* 15827, *K*. *pneumoniae* 1158, respectively, then cloned into pET‐28a (Novagen) for generating a fusion protein PPX with an N–terminal (His)_6_ tag. For the construction of the *drppx*, *ecppx*, and *abppx* expression plasmid, the genes were digested using *Nde*I and *Bam*HI, while the *kpppx* was digested by *Nhe*I and *Bam*HI. For the construction of the *drppx* (NTD) expression plasmid, the *drppx* deleted in the C‐terminal fragment (Δ320–515) was generated by PCR and cloned into pET‐28a following digestion using *Nde*I and *Bam*HI to generate plasmid P‐*drppx* (NTD). Strains and plasmids used in this study are listed in Table S2 (Supporting Information)

Site mutation variant plasmids were constructed according to the procedure described previously.^[^ ^32^ ^]^ Primers used in this study are listed in Table S2 (Supporting Information). Mutations in plasmids were confirmed by DNA sequencing. Three DrPPX mutants, including DrPPX (+7AA), DrPPX (−5AA) and DrPPX (−8AA), were constructed by adding 7 amino acids (VQTFSLA, which was cloned from the α‐helix sequence from the α‐linker of DrPPX), shortening by deletion of 5 amino acids (TFSLA), or shortening by deletion of 8 amino acids (QVQTFSLA) in the terminus of the ɑ‐linker, respectively. The gene *drppx* with an extended or truncated α‐helix sequence, including *drppx*(+7AA), *drppx*(−5AA), *drppx*(−8AA), was synthesized in Tsingke Co., Ltd. The DrPPX mutant genes were ligated into pET‐28a (Novagen).

All expression plasmids were transformed into *E.coli* BL21 Rosetta plysS (DE3) strain, respectively. Cell cultures grown in an LB medium were added with 100 µm IPTG to induce protein expression. Cell pellets separated using centrifuge were resuspended in lysis buffer A (20 mm Tris‐Cl (PH 7.5), 500 mm NaCl, 5% (v/v) glycerol, 10 mm β‐mercaptoethanol, 1% (v/v) Triton X‐100). Cells were lysed using a high‐pressure cell cracker. Following ultracentrifugation (15 000 g, 30 min, 4 °C), the obtained protein supernatant was loaded onto a HisTrap HP column (GE Healthcare, United States), eluted with elution buffer B (20 mm pH 7.5 Tris–HCl, 500 mm NaCl, and 500 mm imidazole). The collected protein fractions were further purified on a Superdex 200 10/300 GL column (GE Healthcare) with elution buffer C (20 mm pH 7.5 Tris–HCl, 250 mm NaCl). Fractions containing the target proteins were pooled, concentrated, flash‐frozen in liquid nitrogen, and stored at −80 °C.

### Protein Crystallization and Structure Determination

Protein crystallization trials were carried out by the sitting drop vapor diffusion method at 20 °C. The protein concentration was 1–5 mg mL^−1^. Crystals of native DrPPX and DrPPX mutant (E114A) were grown in a well containing 0.1 m sodium acetate (NaAc, PH 4.5) and 1.4 m ammonia sulfate (NH_4_SO_4_). The polyP (P5)‐DrPPX and polyP (P2)‐DrPPX complex crystals were grown in the same buffer conditions by the addition of 200 µg mL^−1^ polyP (P20) in the presence of 2 mm Mg^2+^and 8 mm Mn^2+^, respectively. Crystals of DrPPX complex with Pi were grown in a well containing 1.26 m sodium phosphate monobasic monohydrate (NaH_2_PO_4_) and 0.14 m Potassium phosphate dibasic (K_2_HPO_4_), pH 5.6. DrPPX mutant (NTD) crystal was grown in a well containing 3 m sodium acetate (NaAc), PH 7.5. KpPPX crystal was grown in a well containing 0.2 m potassium chloride, 0.05 m HEPES pH 7.5, 35% v/v Pentaerythritol propoxylate (5/4 PO/OH). AbPPX crystal was grown in a well containing 0.2 m ammonium acetate, 0.1 m BIS‐TRIS pH 5.5, 25% w/v polyethylene glycol 3350. Cryocooling was achieved by stepwise soaking the crystals in a reservoir solution containing 10, 20, and 30% (v/v) glycerol for 3 min and flash freezing in liquid nitrogen. Diffraction intensities were recorded on beamline BL17U at Shanghai Synchrotron Radiation Facility (Shanghai, China) and were integrated and scaled with the XDS suite. All the structures of PPXs were determined by molecular replacement using *E. coli* PPX (PDB ID:1U6Z) as the search model.^[^ ^14^ ^]^ Structures were refined using PHENIX.^[^ ^38^ ^]^ and interspersed with manual model building using COOT.^[^ ^39^ ^]^ The statistics for data collection and refinement are listed in Table S1 (Supporting Information). All structural figures were rendered using PyMOL (http://www.pymol.org/).

### Exopolyphosphatase Assay

Exopolyphosphatase assay was performed as described previously with modifications.^[^ ^14^ ^]^ PolyP (P100) and (P20) were gifts from Dr. Adolfo Saiardi. Exopolyphosphatase activity of wild‐type and mutants of PPX was assayed by using the EnzChek Phosphate Assay Kit (ThermoFisher Scientific, Waltham, MA, USA). In the presence of Pi, the enzyme purine nucleoside phosphorylase (PNP) converts 2‐amino‐6‐mercapto‐7‐methylpurine riboside (MESG) to ribose 1‐phosphate and 2‐amino‐6‐mercapto‐7‐methylpurine. The reaction causes a spectrophotometric shift in the maximum absorbance of the substrate from 330 to 360 nm for the product. The exopolyphosphatase reaction was set up by adding P100 into 0.2 mm MESG and 1 U PNP in 20 mm Tris‐NaCl (pH 7.5) with metal ions. The reaction was initiated by the addition of 2 µg PPX or the mutants, then the mixture solution was diluted appropriately after the reaction and measured at 360 nm by Spectrophotometer (SpectraMax M5). The initial slopes were then determined in terms of absorbance/minute and the converted Pi per second was calculated from the phosphate standard curve. To determine the kinetics of each enzyme, the unit of activity was measured for a range of substrate concentrations (1–400 µm) at the enzyme concentration (1–10 µm). The reactions were carried out at 37 °C for 10 min and were independently repeated at least three times. The result was fitted to the Michaelis–Menten equation using PRISM software (GraphPad, San Diego, CA, USA) to determine the *K*_m_ and *k*_cat_ values.

For PAGE gel analysis of exopolyphosphatase activity, the 20 µL reaction mixture includes 100 µm polyP (P100), 2 mm MgCl_2_, and 2 µg PPX protein. Then the reaction was performed in the presence or absence of metal ions at 37 °C. Reaction products were electrophoresed on a 12% (wt./vol) urea–polyacrylamide gel supplied with 7 m urea and stained with DAPI as described previously.^[^ ^35^ ^]^

### Molecular Docking

The docking studies were performed using the AutoDockVina algorithm.^[^ ^40^ ^]^ Prior to docking studies, the ligand and receptor molecules were preprocessed using AutoDock Tools. The grid file was shaped with a grid size of 40  ×  40  ×  40 Å and grid point spacing of 0.375 Å. The docking steps were processed using Lamarckian Genetic Algorithm. The ligands were then ranked by energy values produced during the docking analysis.

### Molecular Dynamics Simulations

Following similar protocols utilized in the previous works.^[^ ^41^, ^42^ ^]^ All‐atom MD simulations were carried out with the software package GROMACS (version 5.1.4).^[^ ^43^ ^]^ The CHARMM36 force field^[^ ^44^, ^45^, ^46^ ^]^ was used for the DrPPX protein, polyP ligand, and ions. The TIP3P model^[^ ^45^ ^]^ was chosen for water molecules. The periodic boundary conditions were applied in all three dimensions. The long‐range electrostatic interactions were computed with the particle mesh Ewald (PME) method^[^ ^48^ ^]^ and the vdW interactions were truncated with a cutoff distance of 1.2 nm. The LINCS algorithm was adopted to constrain the bond vibrations involving hydrogen atoms^[^ ^49^ ^]^ allowing a time step of 2 fs. The temperature T = 300 K was controlled using the velocity‐rescaled Berendsen thermostat.^[^ ^50^ ^]^ The pressure was set to 1 atm using an isotropic Parrinello–Rahman pressostat.^[^ ^51^ ^]^ After the equilibration of the simulation system, each production run was carried out in the NPT ensemble. The obtained MD trajectories were rendered by the VMD program for further analysis.^[^ ^52^ ^]^

To evaluate the structural stability of the protein, the root‐mean‐square deviation was calculated.$\mathrm{RMSD}\;=\sqrt{\frac{1}{N}\sum_{i=1}^{N}{\left[r_{i}\left(t\right)-r_{i}\left(\mathrm{init}\right)\right]}^{2}}$, where *N* is the total number of atoms analyzed, *r_i_* (*t*) is the position of atom *i* at time *t*, and *r_i_* (init) is the initial position of atom *i*. Moreover, the strength of the residue fluctuation was estimated by calculating the root‐mean‐square fluctuation, $\mathrm{RMSF}\;=\;\sqrt{\frac{1}{T}\sum_{t_{j}=\;1}^{N}{\left[r_{i}\left(t_{j}\right)-r_{i}\left(\mathrm{aver}\right)\right]}^{2}}$, where *T* is the simulation time, *r_i_* (*t_j_*) is the position of atom *i* at time *t_j_*, and *r_i_* (aver) is the time‐averaged position of atom *i*. For a certain residue, the mean value of the RMSFs of the atoms it contains was calculated. In Figure 4d, the center of the mass distance between two loops was analyzed separately for four variants (NTD, WT, −5AA, and −8AA). For each variant, the last 100 ns (101 data points) of three independent trajectories were considered, with a total of 303 data points available for analysis. In Figure 4e, the atom contact numbers between NTD and CTD were analyzed for three variants (WT, −5AA and −8AA). For each variant, the last 100 ns (1001 data points) of three independent trajectories were considered, with a total of 3003 data points available for analysis. In Figure 4g, the atom contact numbers between polyP and loops were analyzed for four variants (NTD, WT, −5AA, and −8AA). For each variant, the last 100 ns (101 data points) of three independent trajectories were considered, with a total of 303 data points available for analysis. The Origin Pro software (version 2023b, OriginLab Corporation, MA, USA) was used to visualize the data as a “Box Chart” in Figure 4d,g.

### Differential Scanning Calorimetry (DSC)

Measurements were conducted on a differential scanning calorimeter (STAR System DSC I, Mettler Toledo, Australia). A dry sample (0.04 mg) of protein was loaded in an aluminum tray with 4 µL of distilled water and sealed hermetically. The DSC was calibrated using indium (In^+3^) after a reference point was set up with an empty aluminum container. The trays with the samples were heated at a speed of 5 °C min^−1^ from 20 to 120 °C. The thermogram was constructed ranging from the initial temperature to 120 °C. The melting temperature (*Tm*) and the enthalpy of fusion (*ΔH*) were calculated. An empty tray of the same type was used as a reference.

### Multiple Sequence Alignments and Phylogenetic Analysis

The phylogenetic tree of PPX proteins (42 species in Deinococcus‐Thermus) was constructed by the maximum likelihood method using MEGAX. The unrooted phylogenetic tree was finally visualized using iTOL (https://itol.embl.de/), and the taxonomy was annotated accordingly. To examine the conserved amino acids in the interdomain linker, a multiple protein sequence alignment of PPX proteins (42 species in Deinococcus ‐Thermus) was performed using ClustalW. To examine the conserved amino acids in the polyP binding area of the N‐terminus, a multiple protein sequence alignment of PPX proteins (124 species, covering nearly all phylum of bacteria) was performed. The alignment results were presented using WebLogo (http://weblogo.berkeley.edu/logo.cgi). The structures of DrPPX variants and PPXs from other species were predicted using AlphaFold 2.^[^ ^53^ ^]^

### Statistical Analysis

Unless otherwise stated, data were analyzed using GraphPad PRISM 7.0 (GraphPad Software, San Diego, USA) and were presented as mean ± standard deviation (SD) or mean values. For experimental data, each experiment was performed independently at least three times.

## Conflict of Interest

The authors declare no conflict of interest.

## Author Contributions

S.D., B.W., and R.Y. contributed equally to this work. B.T., R.Z., Y.Z., Y.H., and S.D. were responsible for the experimental design. S.D., B.W., Z.X., N.Y., C.H., J.Z., C.C., and F.Z. performed the experiments. R.Y., R.Z., and D.Z. performed molecular dynamics simulations and analyses. S.D., B.T., R.Z., Y.Z., and Y.H. performed data analysis and drafted the manuscript. All authors read and approved the version to be published.

## Supporting information

Supporting Information

## Data Availability

The data that support the findings of this study are available from the corresponding author upon reasonable request.
