# Supplementary material for: Structural Evolution of Bacterial Polyphosphate Degradation Enzyme for Phosphorus Cycling
Source: Adv Sci (Weinh). 2024 Apr 29;11(26):2309602. doi: 10.1002/advs.202309602 (PMC11234463; doi:10.1002/advs.202309602)
Supplement: Supplementary file 1 — Supporting Information [file ADVS-11-2309602-s001.pdf]

ADVANCED SCIENCE
Open Access

Supporting Information

for *Adv. Sci.*, DOI 10.1002/advs.202309602

Structural Evolution of Bacterial Polyphosphate Degradation Enzyme for Phosphorus Cycling

Shang Dai, Binqiang Wang, Rui Ye, Dong Zhang, Zhenming Xie, Ning Yu, Chunhui Cai, Cheng Huang, Jie Zhao, Furong Zhang, Yuejin Hua, Ye Zhao*, Ruhong Zhou* and Bing Tian**

Supporting Information

Structural Evolution of Bacterial Polyphosphate Degradation Enzyme for Phosphorus Cycling

Shang Dai[1,2#], Binqiang Wang[1,3,4#], Rui Ye[5#], Dong Zhang[1,5], Zhenming Xie[1], Ning Yu[1], Chunhui Cai[1], Cheng Huang[1], Jie Zhao[1], Furong Zhang[1], Yuejin Hua[1,6]*, Ye Zhao[1,6]*, Ruhong Zhou[1,2,5,6]*, Bing Tian[1,6]*

1. Institute of Biophysics, College of Life Sciences, Zhejiang University, Hangzhou, China; 2. Shanghai Institute for Advanced Study of Zhejiang University, Shanghai, China; 3. State Key Laboratory of Clean Energy Utilization, Zhejiang University; 4. Zhejiang Baima Lake Laboratory Co., Ltd; 5. School of Physics, Institute of Quantitative Biology, Zhejiang University, Hangzhou, China; 6. Cancer Center, Zhejiang University, Hangzhou, China

[#] These authors contributed equally to this work.

*Corresponding author: Bing Tian, Ruhong Zhou, Ye Zhao, Yuejin Hua
Email: tianbing@zju.edu.cn; rhzhou@zju.edu.cn; yezhao@zju.edu.cn; yjhua@zju.edu.cn

Keywords: polyphosphate, exopolyphosphatase, structural evolution, α-linker

Supporting Information Text

Molecular Docking and Molecular Dynamics Simulation Analysis

Three docked conformations with the highest scorings were selected, including the Mg^{2+} located in proximity to αP, βP, and an intermediate position between αP and βP of polyP, respectively. These were defined as conformation 1 (Fig S7a), conformation 2 (Fig S7b), and conformation 3 (Fig S7c), respectively. To identify the stable binding conformation of the Mg^{2+} ion, two independent simulations were performed for each of the three conformations. Figure S8a-b showed the conformational change of polyP relative to DrPPX. The motion of polyP with respect to DrPPX was indicated using relative RMSDs. Figure S8a illustrated that polyP in conformation 2 exhibits the smallest relative RMSDs (~0.25nm) compared to the other two conformations (> 0.35nm). This suggests that polyP in the binding pocket is most stable in conformation 2. Additionally, polyP and Mg^{2+} ion in conformations 1 and conformations 3 undergo significant positional adjustments relative to the initial conformation (Fig S8b), whereas the adjustments are smaller in conformation 2, which was consistent with the trend observed in the relative RMSDs (Fig S8a).

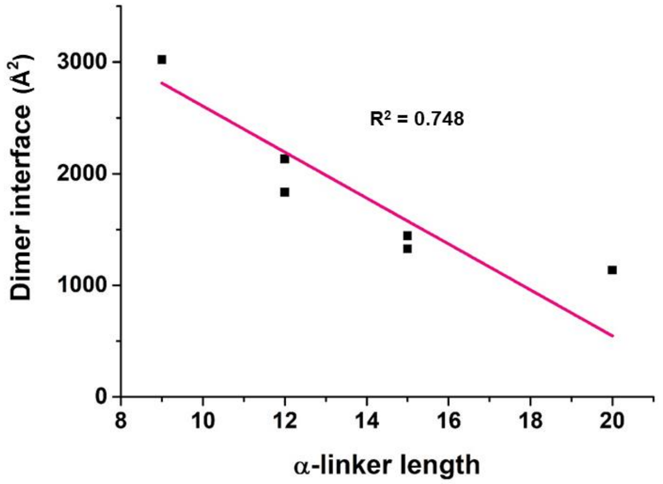

Figure S1. Regression analysis of the correlation between dimer interface and α-linker length.

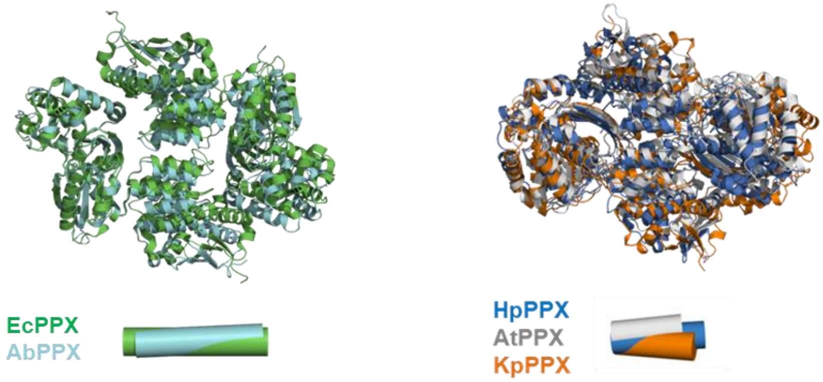

Figure S2. Comparison of the PPX structures containing α-linker with similar length. Protein crystal structures of AbPPX from *Acinetobacter baumannii*, and KpPPX from *Klebsiella pneumoniae* were determined in this work. EcPPX (PDB ID code 1U6Z) from *Escherichia coli*, HpPPX (PDB ID code 6PC0) from *Helicobacter pylori* and AtPPX (PDB ID code 3HI0) from *Agrobacterium tumefaciens* are used for comparison. In the lower panels, the α-linkers in PPXs are highlighted as cylindrical helices with different colors.

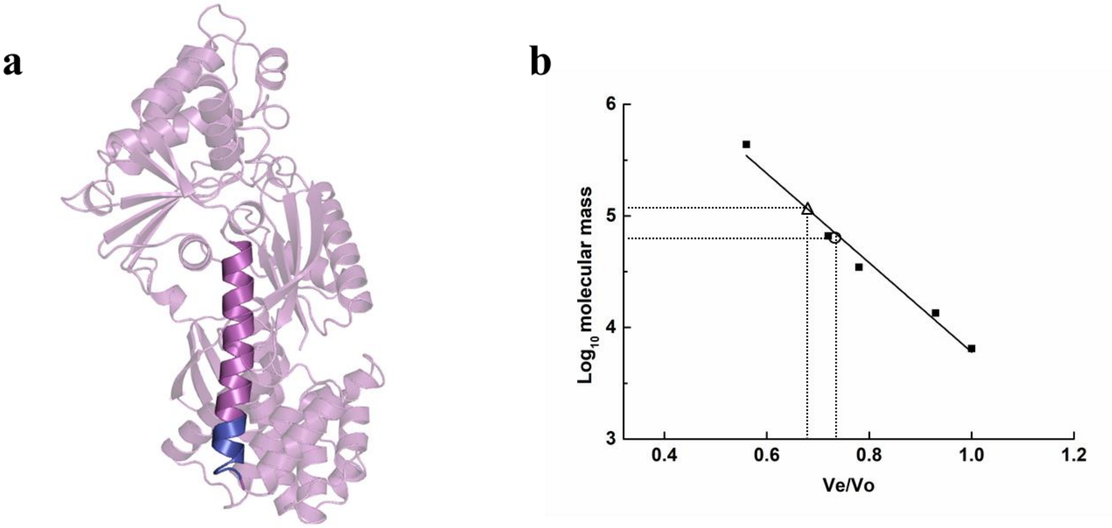

Figure S3. The monomer structure of DrPPX (+ 7 AA) predicted by Alphafold 2 (a) and the relative molecular masses of the DrPPX and DrPPX (+ 7 AA) (b). (a) The extended α-linker in the DrPPX (+ 7AA) was shown in blue. (b) The relative molecular masses of the DrPPX and

DrPPX (+ 7 AA) were calculated by the mathematical relations of molecular mass and elution volumes from a standard elution curve. The standard elution curve (squares) was generated from the elution volumes of the standards divided by 20 ml (Ve/Vo) plotted against the log of the molecular mass of the standards. The elutions of DrPPX (open triangle) and DrPPX (+ 7 AA) (open circle) are shown. The relative molecular mass of DrPPX (124.8 kDa; Ve/Vo =0.67) is 1.85 fold higher than the relative molecular mass of the DrPPX (+ 7 AA) (67.3 kDa; Ve/Vo = 0.74).

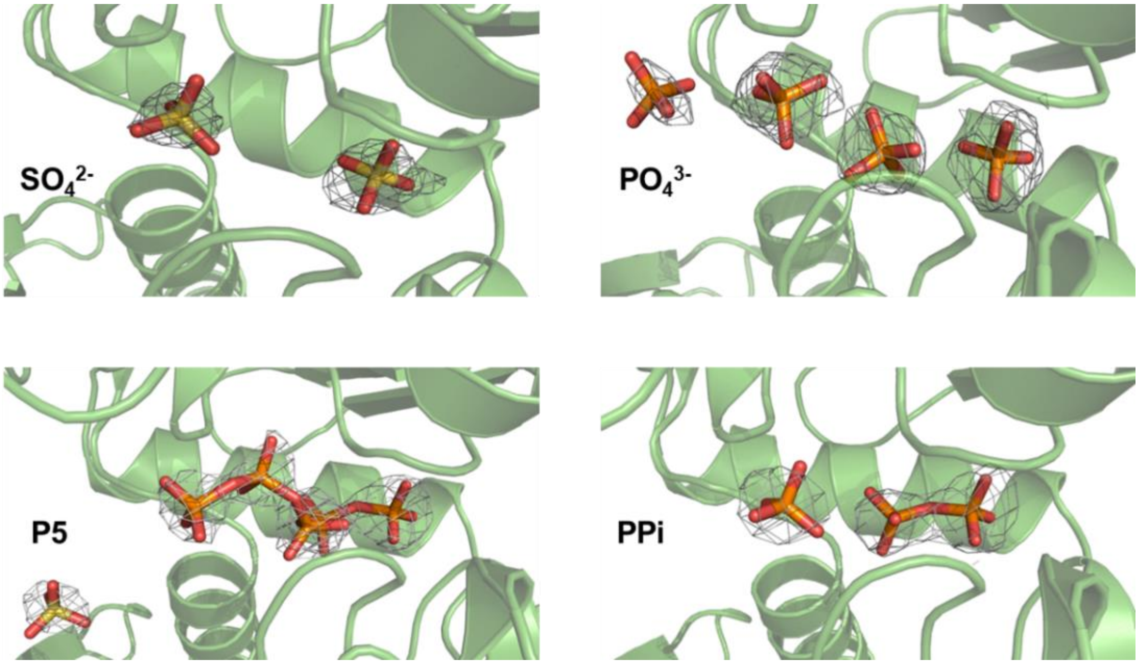

Figure S4. Crystal structures of DrPPX in complex with SO_4^{2-}, PO_4^{3-}, P5 (polyP, n=5) and pyrophosphate (PPi), respectively.

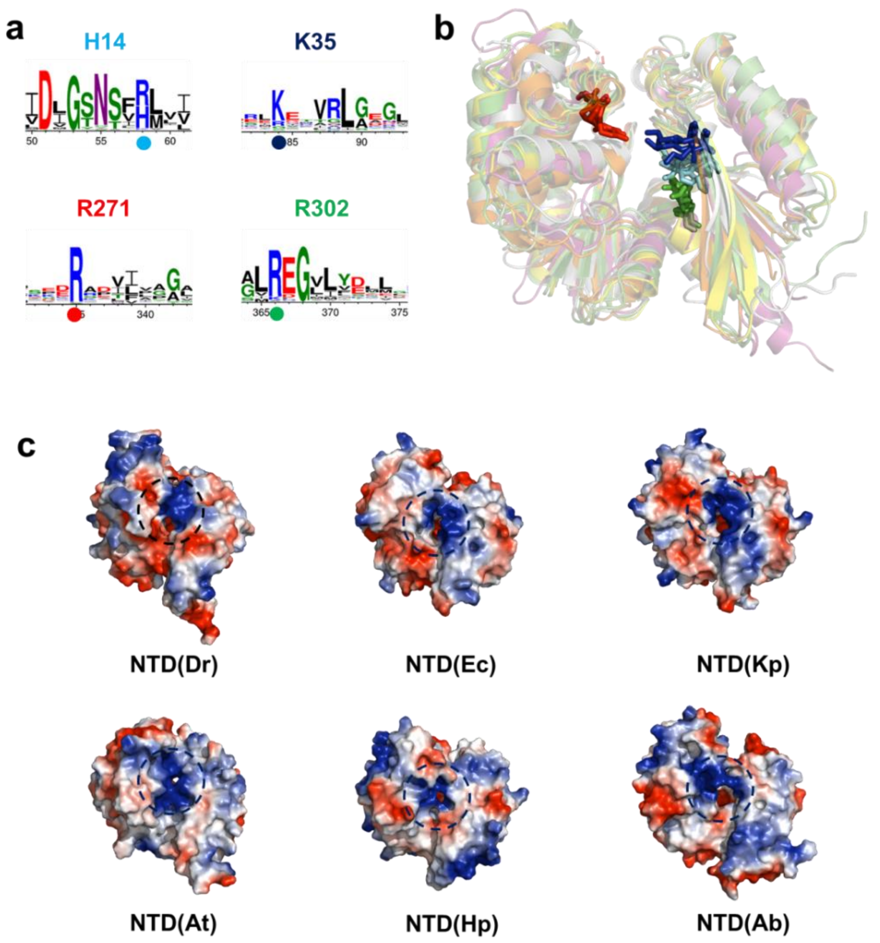

Figure S5. Conserved positively charged residues of PPXs. (a) The conserved positively charged residues were analyzed and presented using WebLogo. The conserved amino acids which correspond to H14, K35, R271 and R302 in DrPPX, were indicated by cyan, blue, red and green dot, respectively. (b) Superposition of NTDs of PPXs from different bacterial species. magentas: DrPPX; yellow: KpPPX; green: EcPPX (PDB ID code 1U6Z); gray: AtPPX (PDB ID code 3HI0); Cyan: AbPPX; orange: HpPPX(PDB ID code 6PC0). The conserved amino acids which correspond to H14, K35, R271 and R302 in DrPPX, were showed as cyan, blue, red and green stick, respectively. (c) Electrostatic potential of N-terminus of PPX calculated using APBS, showing regions of positive potential within the polyP binding cleft. The cleft was indicated by black doted circle.

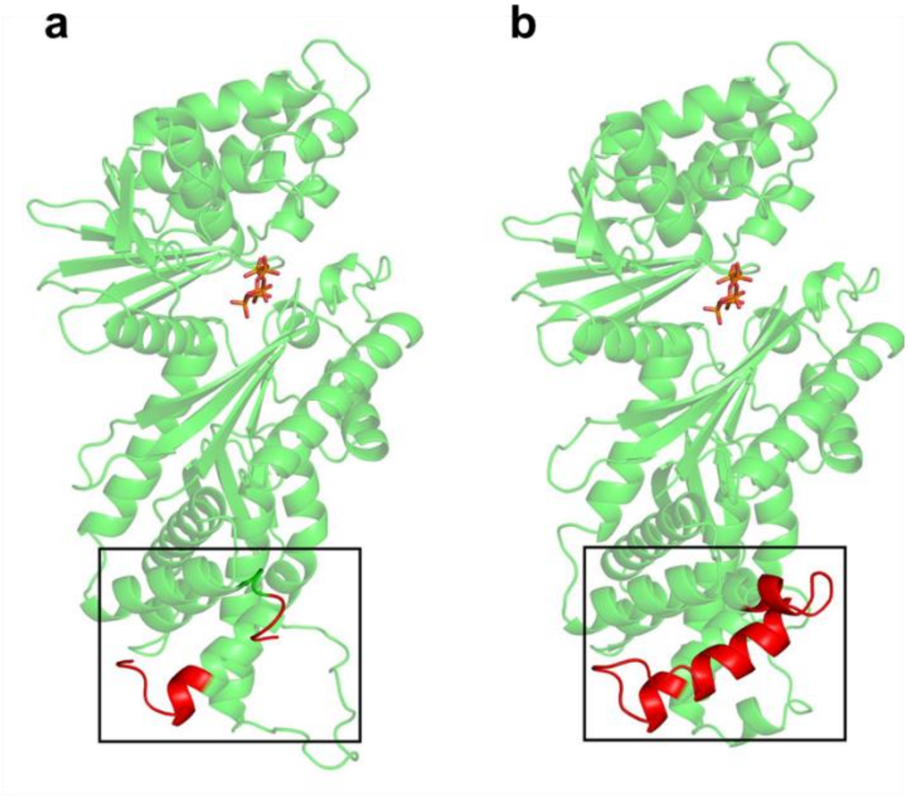

Figure S6. The crystal structure (a) and Alphafold 2 predicted structure (b) of DrPPX. The red part in the black box was the missing region in crystal structure.

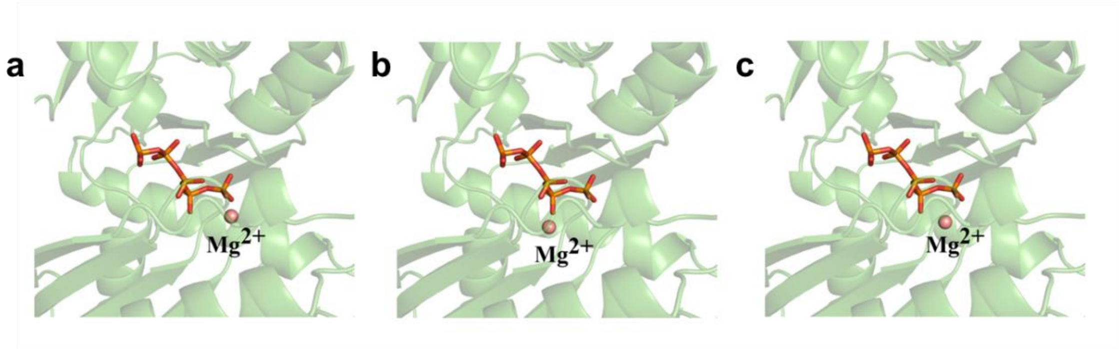

Figure S7. The highest-scoring docked conformations. (a) Mg^{2+} ion close to αP of polyP. (b) Mg^{2+} ion close to βP of polyP. (c) Mg^{2+} ion located between αP and βP of polyP.

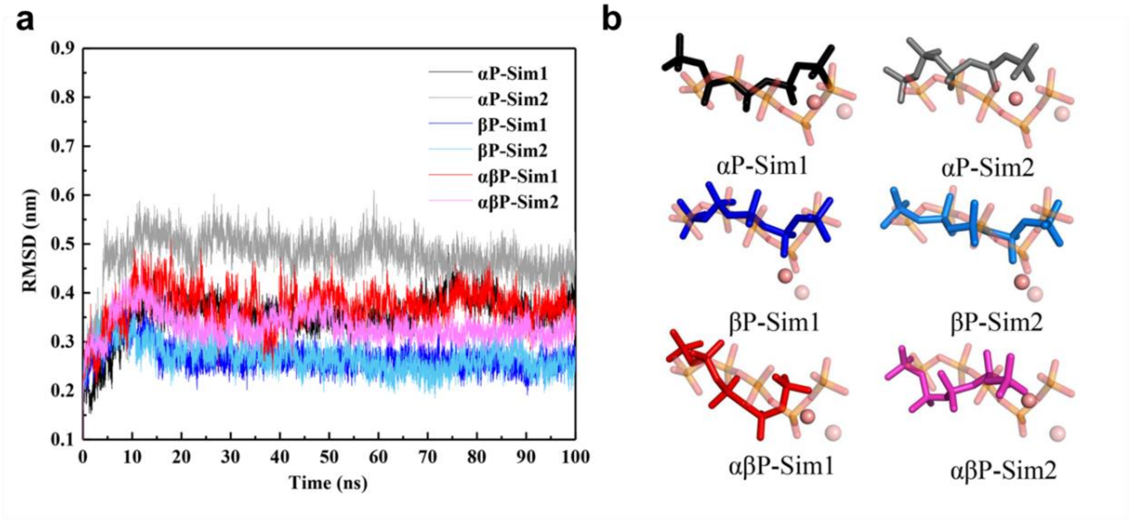

Figure S8. The relative RMSD of the polyP with respect to the PPX (i.e the motion of polyP with respect to PPX protein) in the three conformations of Mg^{2+} ions docking (shown in Fig. S7) (a) and structural comparison of the initial and final interaction of polyP and Mg^{2+} ions (b). Sim1, simulation 1; Sim2, simulation 2. The initial structures were show in shadow.

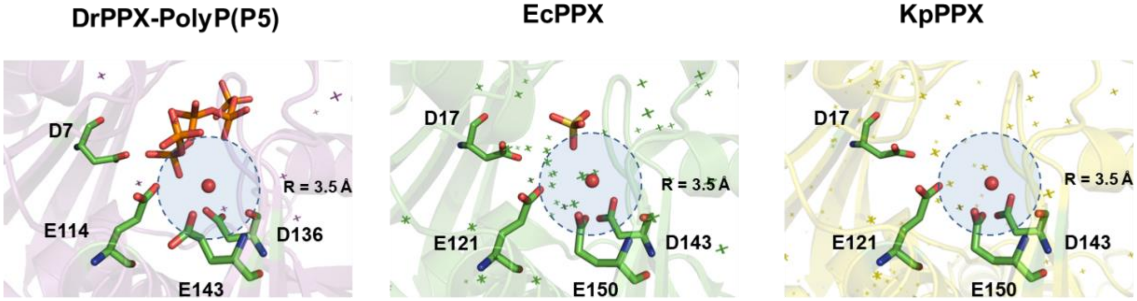

Figure S9. Interaction between three conserved acidic residues (E114, D136 and E143 in DrPPX; E121, D143 and E150 in EcPPX (PDB ID: 1U6Z) and KpPPX) and a water molecule.

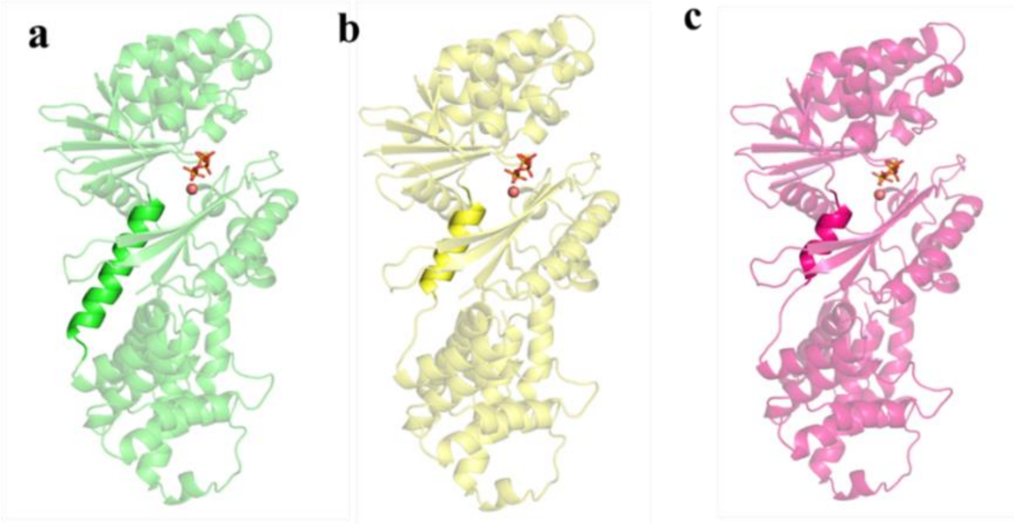

Figure S10. Simulation of initial stuctures of DrPPX mutants with shortened α-linker lengths based on DrPPX wild type as a template. (a) DrPPX wild type; (b) -5AA, DrPPX mutants with deletion of TFSLA in the α-linker; (c) -8AA, DrPPX mutants with deletion of QVQTFSLA in the α-linker. The structures of -5AA and -8AA were predicted by Alphafold 2 using DrPPX wild type as a template. The α-linkers were highlighted.

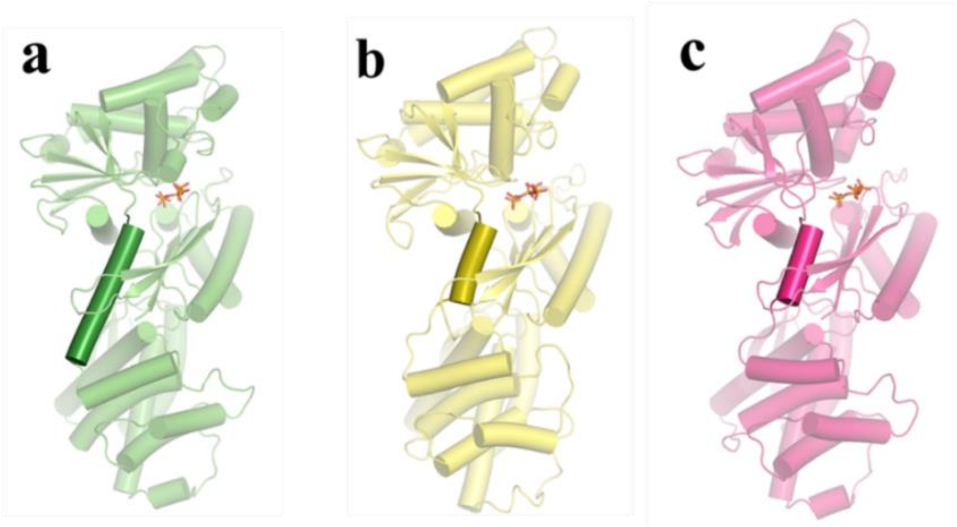

Figure S11. Simulation of the final structures of DrPPX wild type and mutants with shortened α-linker lengths. (a) DrPPX wild type; (b) -5AA, DrPPX mutants with deletion of TFSLA in the α-linke; (c) -8AA, DrPPX mutants with deletion of QVQTFSLA in the α-linker. The α-linkers were highlighted.

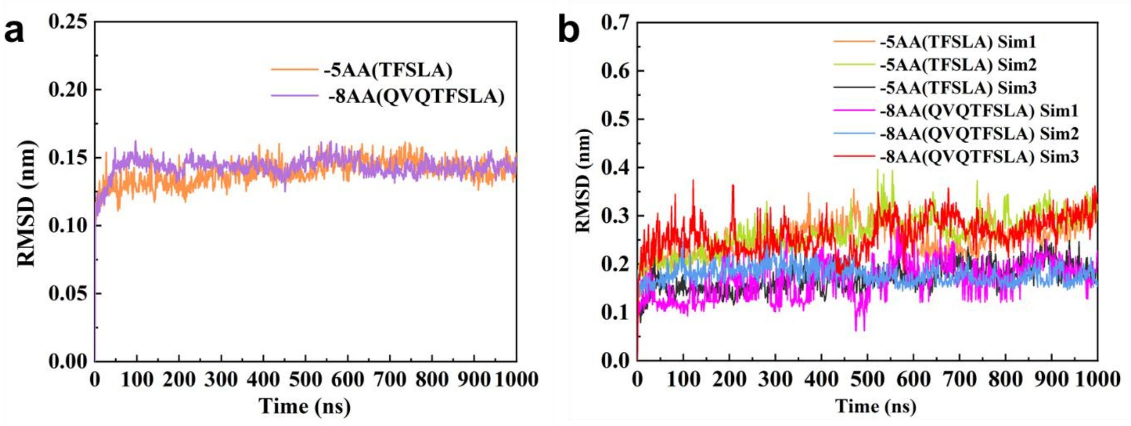

Figure S12. RMSD of the DrPPX variants (-5AA- and -8AA). (**a**) C-terminal and N-terminal domains were imposed with distance restrictions. (**b**) Remove distance restrictions. Three independent simulations (Sim1, Sim2, Sim3) were conducted for each protein structure.

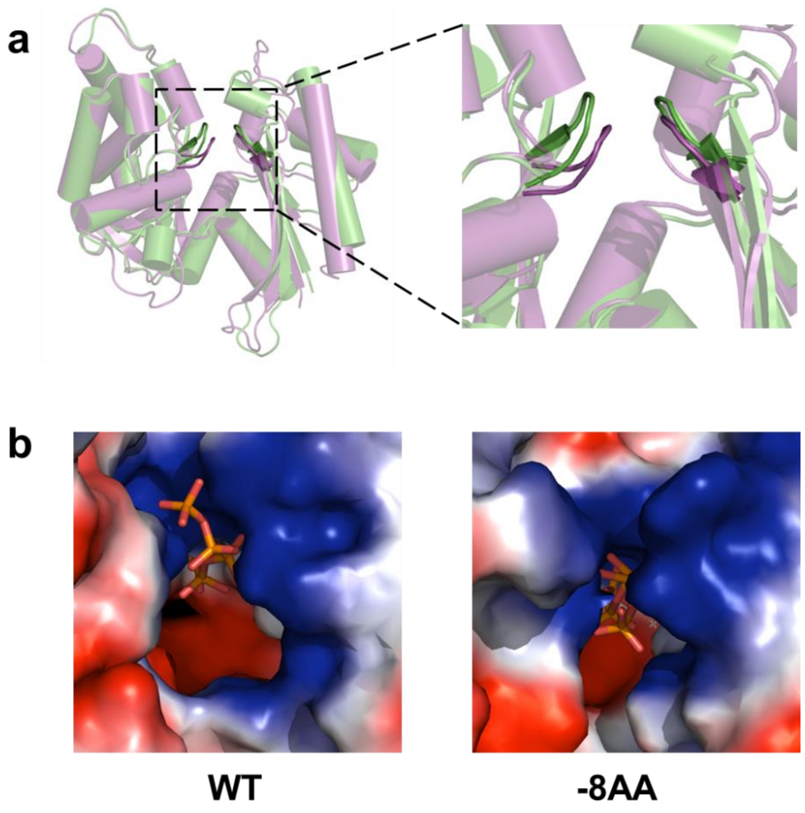

Figure S13. Structural comparison of DrPPX wild type (WT) and DrPPX variant (-8AA) based on molecular dynamics simulation. (a) The left panel shows a superposition of WT (green) and -8AA (magentas). The right panel shows a close-up view of the polyP binding pocket. Loop1 and Loop2 are highlighted. (b) Orientation of WT and -8AA with the polyP-binding pocket. PolyP is represented by stick.

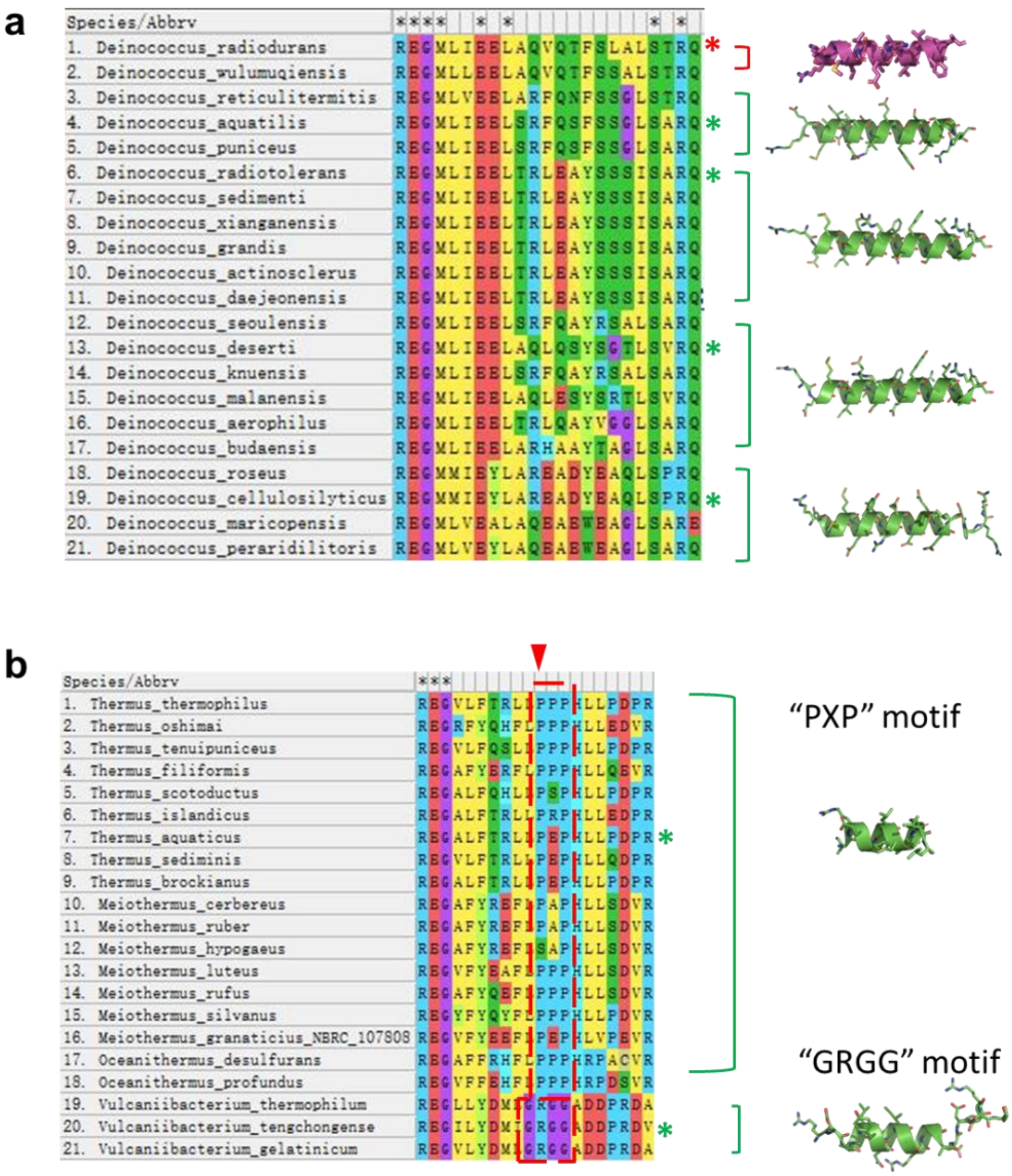

Figure S14. Sequence alignment and structure prediction of α-linker in PPXs. The structures of PPX were predicted using Alphafold 2, and the α-linker were extracted for analysis. *, indicated the PPX which was chosen for structure prediction.

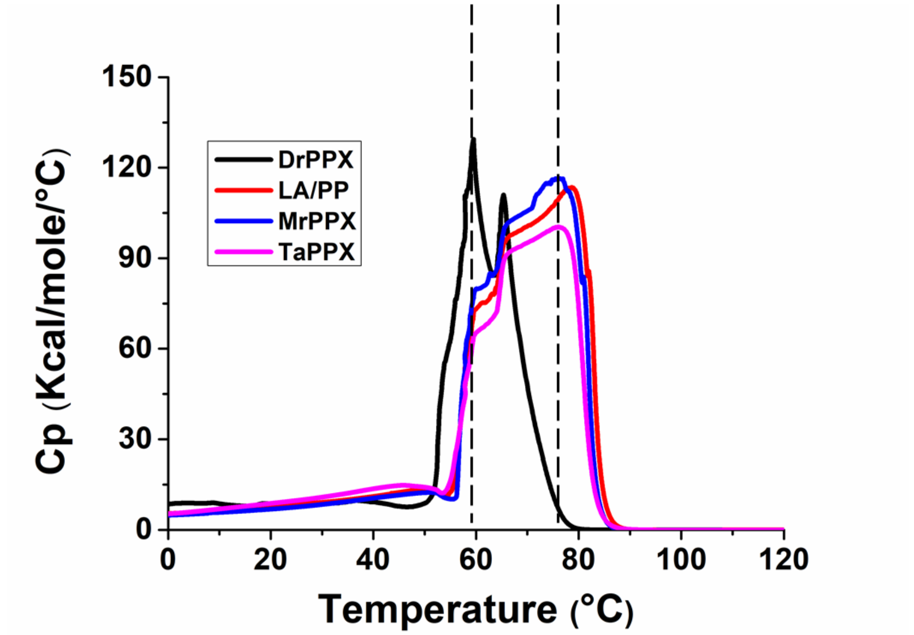

Figure S15. Thermal denaturation curves for DrPPX, DrPPX(LA/PP), MrPPX, and TaPPX.

Table S1: Data collection and refinement statistics of PPX structures

PDB code	**DrPPX** 8JGO	**DrPPX (E114A)** 8JGT	**KpPPX** 8JGW	**AbPPX** 8JGX
**Data collection**				
Space group	$P\ 6_1$	$P\ 6_1$	$P\ 2_12_12_1$	$P\ 2_1$
Cell dimensions				
a, *b*, *c* (Å)	167.07, 167.07, 39.42	164.94, 164.94, 39.21	86.8, 97.08, 144.11	44.22, 160.50, 81.44
α, *β*, *γ* (°)	90.0, 90.0, 120.0	90.0, 90.0, 120.0	90.0, 90.0, 90.0	90.0, 96.3, 90.0
Wavelength (Å)	0.9792	0.9792	0.9792	0.9792
Resolution (Å)	30.0-1.9	30.0-2.2	30.0-1.8	30.0-2.6
R_{sym} (%)	6.0 (36.7)	7.2 (24.4)	5.5 (54.1)	5.3 (55.9)
$I/\sigma I$	16.1 (5.1)	21.7 (5.5)	16.5 (3.4)	15.9 (3.3)
Completeness (%)	98.0 (96.0)	97.5 (86.6)	99.5 (94.5)	97.8 (91.3)
Redundancy	5.3 (5.1)	5.3 (4.5)	6.6 (6.7)	3.7 (3.6)
**Refinement**				
Resolution (Å)	30.0-1.9	30.0-2.2	30.0-1.8	30.0-2.6
No. reflections	49100	30880	113503	33978
$R_{work/}\ R_{free}$	23.1/25.4	23.8/25.7	18.3/20.7	22.5/27.6
No. atoms				
Protein	3619	3567	7943	7613
Ligand/ion	26	21	27	-
Water	186	88	482	-

B-factors				
Protein	45.7	53.1	39.3	76.2
Ligand/ion	59.4	53.3	41.0	-
Water	44.4	44.1	44.6	-
R.m.s deviations				
Bond length (Å)	0.007	0.009	0.006	0.009
Bond angle (°)	0.831	0.976	0.803	1.015
Ramachandran statistics				
Favored (%)	98.1	97.1	98.3	95.9
Allowed (%)	1.9	2.6	1.7	4.1
Outliers (%)	0	0.3	0	0

*Highest resolution shell is shown in parenthesis.

Table S1: Data collection and refinement statistics of PPX structures (continued)

PDB code	**DrPPX-P2** 8JGP	**DrPPX-P5** 8JGQ	**DrPPX-Pi** 8JGR	**DrPPX-NTD** 8JGU
**Data collection**				
Space group	$P\,6_1$	$P\,6_1$	$P\,6_1$	$P\,6_4$
Cell dimensions				
a, *b*, *c* (Å)	168.25, 168.25, 39.84	166.71, 166.71, 39.30	165.01, 165.01, 39.17	101.41, 101.41, 49.26
α, *β*, *γ* (°)	90.0, 90.0, 120.0	90.0, 90.0, 120.0	90.0, 90.0, 120.0	90.0, 90.0, 120.0
Wavelength (Å)	0.9792	0.9792	0.9792	0.9792
Resolution (Å)	30.0-3.0	30.0-2.5	30.0-2.5	30.0-2.0
R_{sym} (%)	9.0 (39.3)	6.9 (52.9)	6.2 (54.8)	10.9 (57.0)
$I/\sigma I$	8.7 (1.3)	11.7 (2.5)	17.7 (3.2)	17.2 (7.2)
Completeness (%)	93.6 (91.7)	94.4 (98.6)	94.4 (96.5)	98.0 (96.5)
Redundancy	3.5 (2.8)	3.0 (3.0)	5.9 (5.9)	7.0 (7.1)
**Refinement**				
Resolution (Å)	30.0-3.0	30.0-2.5	30.0-2.5	30.0-2.0
No. reflections	12059	20798	20444	19612
$R_{work/}$ R_{free}	26.4/31.7	21.2/24.8	22.0/24.5	18.4/21.3
No. atoms				
Protein	3569	3629	3554	2288
Ligand/ion	25	52	22	1
Water	-	32	16	172
B-factors				
Protein	88.9	53.8	68.0	26.0
Ligand/ion	94.2	78.1	57.4	31.6
Water	-	44.2	57.0	30.9
R.m.s deviations				
Bond length (Å)	0.011	0.007	0.007	0.007
Bond angle (°)	1.487	0.901	0.869	0.824
Ramachandran statistics				
Favored (%)	97.3	96.9	97.7	97.1
Allowed (%)	2.7	3.1	2.3	2.9
Outliers (%)	0	0	0	0

*Highest resolution shell is shown in parenthesis.

Table S2. Strains and plasmids used in this study.

Strains or plasmids	Description	Source
**Strains**		
D. radiodurans R1	ATCC 13939	Lab stock
Escherichia coli	Strain K12	Lab stock
Meiothermus ruber	Strain DSM 1279	DSMZ
Thermus aquaticus	Strain DSM 625	DSMZ
**Plasmid**		
pET28a+	Expression vector with strong T7 promotor, His-tag	Lab stock

Table S3. Primers used in this study.

Primer	Sequence (5'- 3')
Protein expression	
DrPPX-P1	GGAATTCCATATGATGCGGGTCGCCGTCG
DrPPX-P2	CGGGATCCTCAGGTGGCTTTGGCCTCTG
AbPPX-P1	GGAATTCCATATGATGTCTGACTTTCTGATTGATGAAG
AbPPX-P2	CGGGATCCTTAATCGATAAATTTTTCCGACTGA
KpPPX-P1	GGAATTCGCTAGCATGCCAATAAACGATAACACCC
KpPPX-P2	CGGGATCCTCAGGCGTCTGGCTCTTCTTCAGCA
EcPPX-P1	GGAATTCCATATGATGCCAATACACGATAAATCCCCTC
EcPPX-P2	CGGGATCCTTAAGCGGCGATTTCTGGTGTACTT
DrPPX(NTD)-P1	GGAATTCCATATGATGCGGGTCGCCGTCG
DrPPX(NTD)-P2	CGGGATCCTCAGGTGGCTTTGGCCTCTGCCG
MrPPX- P1	GGAATTCCATATGGTGCAACGACTGGGCATTGTAGATC
MrPPX- P2	CGGGATCCCTAGCCTCGAGGCCCCCA
TaPPX-P1	GGAATTCCATATGGTGCTACAGATTACGCCAGAGC
TaPPX-P2	CGGGATCCCTACCACGCCACCTCTAACCC
Protein mutation for DrPPX	

DrPPX(T10A)-P1	ACTGGAGTTGGCTCCCACATCGGCGACG
DrPPX(T10A)-P2	CGTCGCCGATGTGGGAGCCAACTCCAGT
DrPPX(N11A)-P1	AGGAGGTGACTGGAGGCGGTTCCCACATCGGC
DrPPX(N11A)-P2	GCCGATGTGGGAACCGCCTCCAGTCACCTCCT
DrPPX(S12A)-P1	TCAGGAGGTGACTGGCGTTGGTTCCCACATC
DrPPX(S12A)-P2	GATGTGGGAACCAACGCCAGTCACCTCCTGA
DrPPX(H14A)-P1	GCAATCAGGAGGGCACTGGAGTTGGTTCCCACAT CG
DrPPX(H14A)-P2	CGATGTGGGAACCAACTCCAGTGCCCTCCTGATTG C
DrPPX(K35A)-P1	GGCGGGTGCGGTCCGCGAGGGTGTCTATGA
DrPPX(K35A)-P2	TCATAGACACCCTCGCGGACCGCACCCGCC
DrPPX(T82A)-P1	GCGCCTCGCGCAGCGCGGAGGCCGCGTAGACGC
DrPPX(T82A)-P2	GCGTCTACGCGGCCTCCGCGCTGCGCGAGGCGC
DrPPX(S141A)-P1	GCACGAATTCCAAAGCGCCGCCGCCGAGGT
DrPPX(S141A)-P2	ACCTCGGCGGCGGCGCTTTGGAATTCGTGC
DrPPX(R271A)-P1	GCGGCGAGGATGGTGTCGCCCGCGCGCTCCAGCC C
DrPPX(R271A)-P2	GGGCTGGAGCGCGCGGGCGACACCATCCTCGCCG C
DrPPX(D7A)-P1	GTTGGTTCCCACAGCGGCGACGGCGAC
DrPPX(D7A)-P2	GTCGCCGTCGCCGCTGTGGGAACCAAC
DrPPX(E114A)-P1	CATCAGCGGCGTACGCGAGGGCGAACTCACCTAC CTC
DrPPX(E114A)-P2	GTAGGTGAGTTCGCCCTCGCGTACGCCGCTGATGA CCG
DrPPX(E136A)-P1	CGCCGCCGAGGGCGAGCAGCACG
DrPPX(E136A)-P2	CGTGCTGCTCGCCCTCGGCGGCG
DrPPX(E143A)-P1	GCCGCGCACGAATGCCAAACTGCCGCC
DrPPX(E143A)-P2	GGCGGCAGTTTGGCATTCGTGCGCGGC
α-linker mutation for DrPPX	
DrPPX(L310P)-P1	GGGATGCTGATCGAGGAACCCGCTCAG
DrPPX(L310P)-P2	GGTTCCTCGATCAGCATCCCTTCG
DrPPX(A311P)-P1	GATGCTGATCGAGGAACTCCCTCAGGTC
DrPPX(A311P)-P2	GGAGTTCCTCGATCAGCATCCCTTC
DrPPX(V313P)-P1	GGAACTCGCTCAGCCCCAGACT
DrPPX(V313P)-P2	GGCTGAGCGAGTTCCTCGATCAGC
DrPPX(Q314P)-P1	GAGGAACTCGCTCAGGTCCCGACTTT
DrPPX(Q314P)-P2	GGGACCTGAGCGAGTTCCTCGATC
DrPPX(S317P)-P1	CTCAGGTCCAGACTTTCCCCTTGGCG
DrPPX(S317P)-P2	GGGAAAGTCTGGACCTGAGCGAGTTCC
DrPPX(L318P)-P1	GTCCAGACTTTCAGCCCGGCGCT
DrPPX(L318P)-P2	GGGCTGAAAGTCTGGACCTGAGCG

Underlines indicate the respective restriction sites
